# Multiple Dimerization Modes in Thiocarboxylate Paddlewheel Complexes: A Comprehensive View of Energy Landscapes from DFT Calculations and Statistics

**DOI:** 10.1002/chem.202503004

**Published:** 2025-12-02

**Authors:** Olga Mironova, Giacomo Bellini, Alessio Nicolini, Andrea Cornia

**Affiliations:** ^1^ Dipartimento di Scienze Chimiche e Geologiche e UdR INSTM Università degli Studi di Modena e Reggio Emilia Modena Italy

**Keywords:** lantern complexes, platinum, polymorphism, solvatomorphism, vanadium

## Abstract

Thiocarboxylate paddlewheels (PWs) [MTr(SOCR)_4_L] (M = Pt, Pd; Tr = first‐row transition metal; L = Tr‐coordinated axial ligand) form a variety of dimeric structures via M···M' and M···S' contacts. We found that [PtVO(SOCPh)_4_] (**1**), a molecular spin qubit, yields three crystalline toluene (tol) solvates, namely **1**·0.875tol and two polymorphic **1**·0.5tol phases. The crystals contain either staggered quasi‐coaxial dimers with short Pt···Pt' distances (3.17‐3.23 Å) or heavily bent noncoaxial molecular pairs supported by Pt···S' contacts (3.34‐3.38 Å). By contrast, in the known solvatomorphs **1**·CH_2_Cl_2_ and **1**·0.5hex (hex = *n*‐hexane), two collinear molecules compose a “square” dimer via a pair of reciprocating Pt···S' contacts (3.13‐3.16 Å). According to gas‐phase DFT calculations (PBE0/def2‐TZVPP/D3BJ), dimerization is energetically favored by 15–20 kcal mol^−1^ and is guided by a shallow potential energy surface, with staggered dimers as ground configurations but eclipsed and square dimers well within energetic reach. Inspection of the local energy minima also disclosed a previously unrecognized eclipsed arrangement with ∼45° twisting of both PWs relative to the metal plane, whose existence was confirmed by statistical analysis of PW structures in the Cambridge Structural Database. Our results led to a new classification scheme for these PW dimers relevant to molecular magnetism and quantum technologies.

## Graphical abstract

Staggered, square, and heavily bent dimers occur in the five known solvatomorphs of paddlewheel (PW) complex [PtVO(SOCPh)_4_]. These and other structural motifs are well within energetic reach due to the shallow potential energy surface that guides dimerization and are included in a revised classification of available structural data on heterobimetallic PWs.

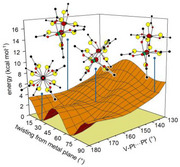

## 1 Introduction

**FIGURE 1:**
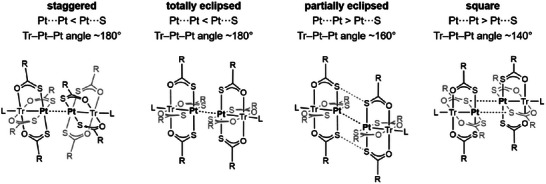
Configurations of dimeric structures formed by PW complexes [PtTr(SOCR)_4_L] (Tr = first‐row transition metal; L = Tr‐coordinated axial ligand). Adapted with permission from Ref. [1]. Copyright 2018 American Chemical Society.

Heterobimetallic paddlewheel (PW) complexes [MTr(SOCR)_4_L] (M = Pt, Pd; Tr = first‐row transition metal; L = Tr‐coordinated axial ligand) display a remarkable variety of supramolecular arrangements in the solid state, with dimeric structures supported by M···M' and M···S' contacts as the most common motif (dots indicate intermolecular contacts) [1]. Over the years, the group of L. Doerrer has introduced and refined a useful and widely adopted classification of these dimers [1, 2]. For M = Pt, three geometrical parameters have been considered (Figure 1): the Pt···Pt' distance, the Tr−Pt···Pt' angle, and the shortest Pt···S' distance. In the *staggered* dimers, the Pt···Pt' separation (∼3.1 Å) is shorter than the Pt···S' distance and is also shorter than the sum of two Pt van der Waals (vdW) radii (3.44 Å) [3]. Such a close metal‐metal contact requires a twisting of the two PtS_4_ moieties by ca. 45° relative to one another (hence the name) and a virtually linear metal arrangement (Tr−Pt···Pt' ∼ 180°), that is, the two PWs in the dimer are *coaxial*. *Totally eclipsed* dimers also feature approximately coaxial PWs, with Pt···Pt' < Pt···S' and a Tr─Pt···Pt' angle close to 180°; however, in this category the smallest S─Pt···Pt'─S' dihedral is close to zero, implying a significantly larger Pt···Pt' separation than in staggered dimers (ca. 3.4 Å). In *partially eclipsed* dimers, the two PWs are no longer coaxial, as the Tr─Pt···Pt' angle is reduced to ∼160°; the two PtS_4_ moieties remain, however, collinear, and the Pt···S' distance decreases below Pt···Pt'. *Square* dimers are similar, but the Tr−Pt···Pt' angles are between 130 and 150°; in this last category, a [Pt_2_S_2_] quadrilateral is formed, with two reciprocating Pt···S' contacts shorter than the sum of the vdW radii for Pt and S (3.0‐3.3 vs. 3.55 Å) [3] and the largest Pt···Pt' distance in the family.

The dimerization mode is not merely of descriptive interest but also impacts some physical properties. For instance, staggered dimers with paramagnetic Tr metals display the largest intradimer superexchange coupling due to the short metallophilic contact [4, 5, 6, 7, 8]. Thanks to the easily interpretable magnetic behavior of a pair of interacting *S* = ½ spins, this trend is especially clear in the family of vanadyl‐based PWs (TrL = VO) [5, 7, 8]. However, within the pool of structurally authenticated compounds, it is often difficult to draw any conclusion on the existence of a preferred dimerization motif because several factors can affect the structure adopted by the dimers: (a) the transition metal Tr; (b) the R substituent on the thiocarboxylate ligands [4]; (c) the apical ligand L on Tr [9]; (d) the solvent used for crystallization and often incorporated in the crystal structure; (e) crystallization rate. In the unique case of [{Na(12‐crown‐4)_2_}{PtTr(SOCMe)_4_(NCS)}]·acetone (Tr = Co, Ni, Zn), monomeric and dimeric PWs even coexist in the same crystal [10].

Typically, varying Tr while otherwise keeping the same composition yields isomorphous crystals, as does changing the heavier metal from Pt to Pd [6, 8]. Instead, the axial ligand and crystallization conditions play a major role. For instance, complex [PtNi(SOCMe)_4_(H_2_O)] is obtained in three solvatomorphic modifications featuring different dimerization modes: staggered (upon slow reaction and crystallization in water, using a W‐shaped tube) [11], totally eclipsed (upon slow evaporation of a MeCN solution) [2], and partially eclipsed (upon cocrystallization with phenazine) [12]. [PtCo(SOCPh)_4_(H_2_O)] behaves similarly, forming staggered dimers in its THF solvate but a square dimeric structure when crystallized from CH_2_Cl_2_ [13]. The role of kinetic factors is exemplified by the solvent‐free complex [PtNi(SOCMe)_4_(pyNO_2_)], which is obtained as eclipsed dimers when fast crystallized from hot, nearly boiling CH_2_Cl_2_ solutions [6], but as staggered dimers upon slow evaporation of a CH_2_Cl_2_:acetone (∼1:4 v/v) mixture (pyNO_2_ = 3‐nitropyridine) [4].

We herein highlight the utmost ductility of dimerization motifs exhibited by the vanadyl‐containing complex [PtVO(SOCPh)_4_] (**1**) [7], which was recently recognized as an electronic spin qubit [5] of interest in the emerging and strongly multidisciplinary field of molecule‐based quantum technologies [14, 15, 16, 17]. Complex **1** was preferred over its Pd analogue [8] because of its greater stability during chemical manipulations. Although EPR spectroscopy and ^1^H NMR DOSY prove it is monomeric in organic solutions [5], **1** forms a variety of solvatomorphs when crystallized from different solvents. The isomorphous crystalline phases **1**·CH_2_Cl_2_ [7] and **1**·0.5hex [18], containing square dimers, are obtained by layering *n*‐hexane (hex) over CH_2_Cl_2_ and THF solutions of crude **1**, respectively. Their crystals suffer from rapid solvent loss upon isolation and storage, which complicates solid‐state measurements such as magnetic characterizations [5]. Searching for alternative crystalline phases more resistant to solvent loss, we attempted crystallization from toluene (tol). This solvent has a considerably higher boiling point than CH_2_Cl_2_ and yields at least two different solvatomorphs: **1**·0.875tol, which contains staggered dimers and represents the dominant phase in crystallization batches, and **1**·0.5tol. This hemi‐toluene solvate further exists in two polymorphic phases, herein denoted as α‐**1**·0.5tol and β‐**1**·0.5tol. The former contains staggered dimers, while β‐**1**·0.5tol features the longest intradimer Pt···Pt' contact in the series and a considerably distorted geometry that does not correspond to any of the structural motifs depicted in Figure 1. The five crystalline phases now available also provide an opportunity to track changes in other geometrical parameters, like the Pt−V and V═O distances, as a function of the packing motif alone. Most importantly, they show that different supramolecular arrangements are accessible even when all the above‐listed factors, including the crystallization solvent, are kept constant. This highlights a very shallow potential energy surface for dimerization [2], which was explored by gas‐phase DFT optimization of experimental geometries and relaxed surface scans. Following these calculations and a statistical analysis of [MTr(SOCR)_4_L] entries in the Cambridge Structural Database (CSD), we finally propose a revision of the current classification scheme, with the addition of a new subcategory of eclipsed dimers.

## 2 Results and Discussion

### 2.1 Synthesis

Crude **1** was recrystallized by storing a suspension in toluene at 60°C. In the course of four days, X‐ray quality crystals of different shapes formed in the vial. The main mass of long, plate‐shaped crystals under the mother liquor was characterized as **1**·0.875tol. Solvent‐deficient modifications with composition **1**·0.5tol formed as deep green rhombus‐shaped and block‐like individuals on the walls of the vial, above the solution level.

### 2.2 X‐ray Crystallography

**FIGURE 2:**
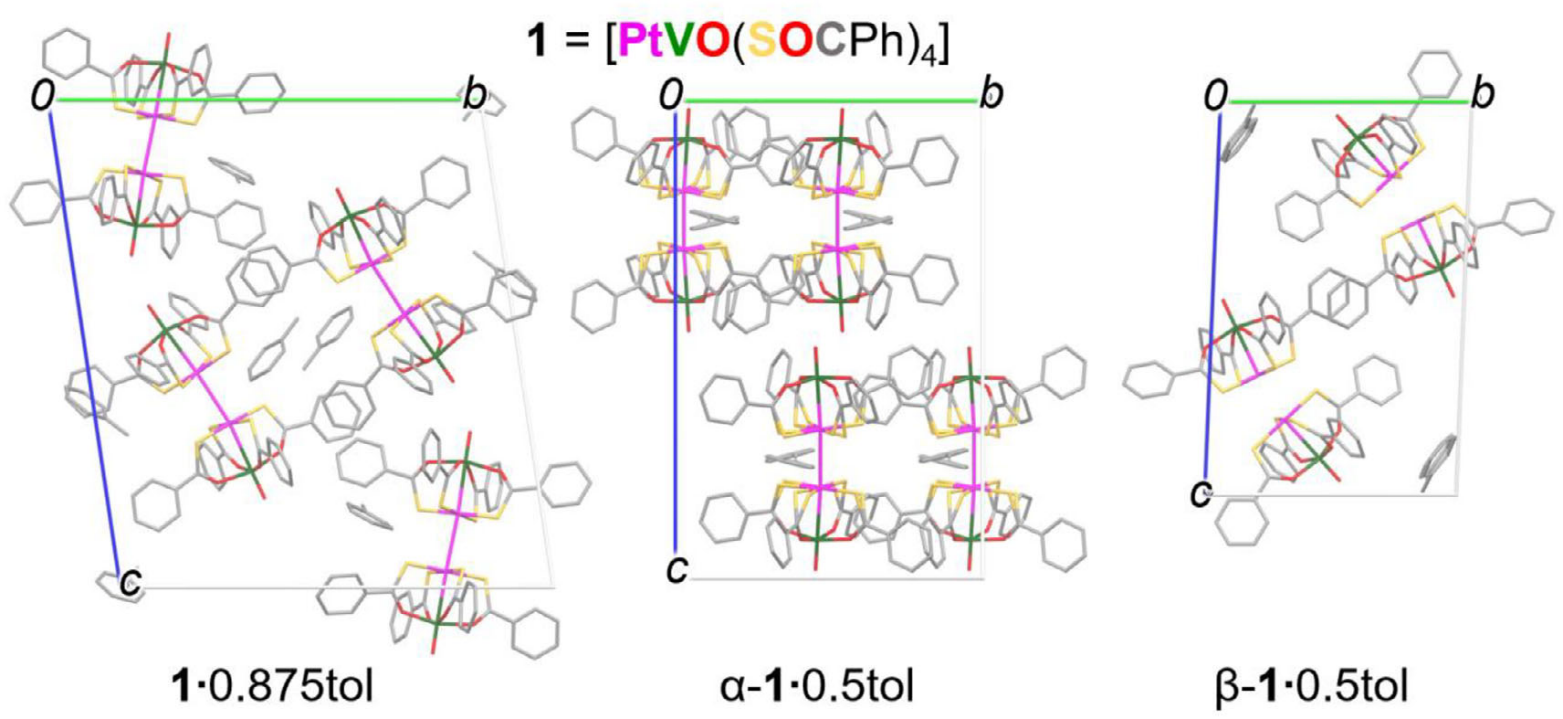
Unit cells of the three crystalline phases obtained from toluene, viewed along the *a* axis and drawn using capped sticks. Hydrogen atoms are omitted for clarity.

**FIGURE 3:**
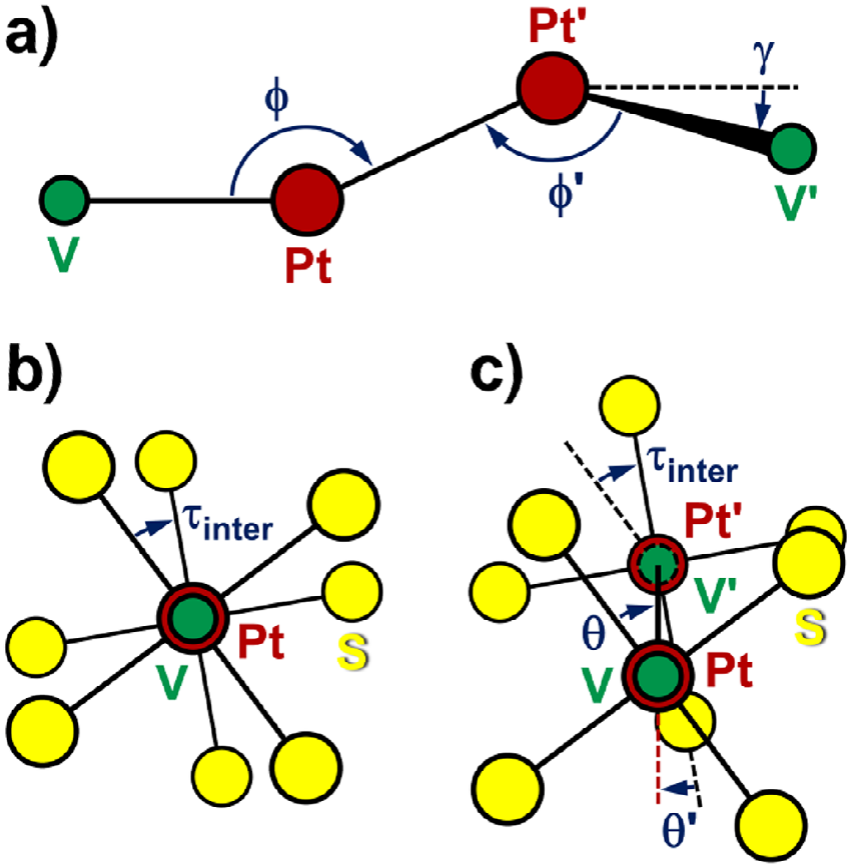
(a) Tetrametallic core of the PW dimers studied in this work, projected onto the V─Pt···Pt' plane, with the atom labels and the definition of the angles ϕ, ϕ', and γ. (b,c) On‐axis views of dimeric structures with collinear V−Pt vectors (i.e., γ = 0 and consequently ϕ = ϕ’), projected in a plane normal to the V−Pt direction. The models include the V, Pt, and S atoms and assume VPtS_4_ moieties with *C*_4v_ symmetry. Panel (b) shows a coaxial dimer with a linear metal array (ϕ = 180°), while panel (c) depicts a noncoaxial dimer (ϕ ≠ 180°). τ_inter_ is the smallest rotation of the front PW unit (depicted using slightly larger spheres) that makes the two PtS_4_ moieties collinear (a positive value indicates a clockwise rotation; |τ_inter_| ≤ 45°). In panel (c), the angles θ and θ' define the orientation of the PtS_4_ moieties with respect to the metal plane. θ is the smallest rotation around the V−Pt bond that brings one of the S atoms of the front PW unit onto that plane (|θ| ≤ 45°). θ’ is defined similarly for the back PW unit. When the dimer is viewed as in this figure, positive values of θ and θ' are associated with clockwise and counterclockwise rotations, respectively, so that θ + θ’ = τ_inter_.

Three crystalline phases were identified in crystallization batches from toluene and investigated by single‐crystal X‐ray diffraction (SXRD), affording the crystal data and refinement parameters gathered in Table S1. Unit cell structures are depicted in Figure 2, while selected structural parameters are presented in Table 1 (necessary definitions are available in the footnotes of Table 1 and in Figure 3). Note that our discussion of torsion angles involving S atoms is limited to PtS_4_ moieties without resolvable rotational disorder. For comparison, in the same table we include data for **1**·CH_2_Cl_2_ [7] and **1**·0.5hex [18], which contain centrosymmetric square dimers, and for the thioacetato derivative [PtVO(SOCMe)_4_] (**2**), which forms staggered dimers with crystallographically imposed *C*_2_ symmetry [7].

**TABLE 1:** Selected bond distances and angles in solvatomorphs of **1**.

		Distances (Å)^a^				Angles (°)^a^			
Compound		V═O	V–Pt	Pt···Pt'	Pt···S' ^b^	V–Pt···Pt' (ϕ)	τ_intra_ ^c^	τ_inter_ ^d^	γ^e^
**1**·CH_2_Cl_2_ (*P* $\bar{1}$)^f^		1.581(4)	2.7823(10)	3.8408(5)	3.1266(14)	143.62(2)	20.6	0.0	0.0
**1**·0.5hex (*P* $\bar{1}$)^g^		1.5855(13)	2.7981(3)	3.9486(2)	3.1612(5)	142.41(1)	17.6	0.0	0.0
**1**·0.875tol (*P* $\bar{1}$)	mol1 mol2 mol3 mol4	1.582(5) 1.577(5) 1.566(5) 1.575(5)	2.8582(14) 2.8256(14) 2.8389(14) 2.8256(13)	3.1744(5) – 3.2342(5) –	3.848(2) 3.827(2) 3.84‐3.87^h^ 3.74^h^	176.62(3) 176.12(3) 170.35(3) 175.06(3)	11.6 16.1 –^h^ –^h^	42.2 – –^h^ –	1.96(5) – 11.87(4) –
α‐**1**·0.5tol (*C*2/*c*)		1.581(2)	2.8321(5)	3.1740(2)	3.84‐3.88^h^	176.381(11)	–^h^	–^h^	6.890(16)
β‐**1**·0.5tol (*P* $\bar{1}$)	mol1 mol2	1.586(2) 1.581(2)	2.7997(5) 2.8138(5)	4.5100(2) –	3.3761(9) 3.3426(8)	135.41(1) 126.84(1)	17.0 16.8	– –	19.311(15) –
**2** (*C*2/*c*)^f^		1.592(2)	2.8635(6)	3.1747(4)	3.8526(9)	177.168(11)	15.2	32.6	0.820(15)

^a^ Dots indicate intermolecular contacts; primed symbols are used for atoms of the neighboring PW in a dimer.

^b^ Shortest Pt···S' contact.

^c^ Intramolecular twisting angle, evaluated as the average O−V−Pt−S dihedral angle (with O and S belonging to the same thiocarboxylate ligand).

^d^ Intermolecular twisting angle; in coaxial or quasi‐coaxial dimers (ϕ ∼ 180°), it is evaluated by averaging the smallest set of S−Pt···Pt'−S' dihedrals (see Figure 3b).

^e^ Smallest angle between the two V−Pt axes (see Figure 3a).

^f^ From Ref. [7].

^g^ From Ref. [18].

^h^ Affected by rotational disorder.

**FIGURE 4:**
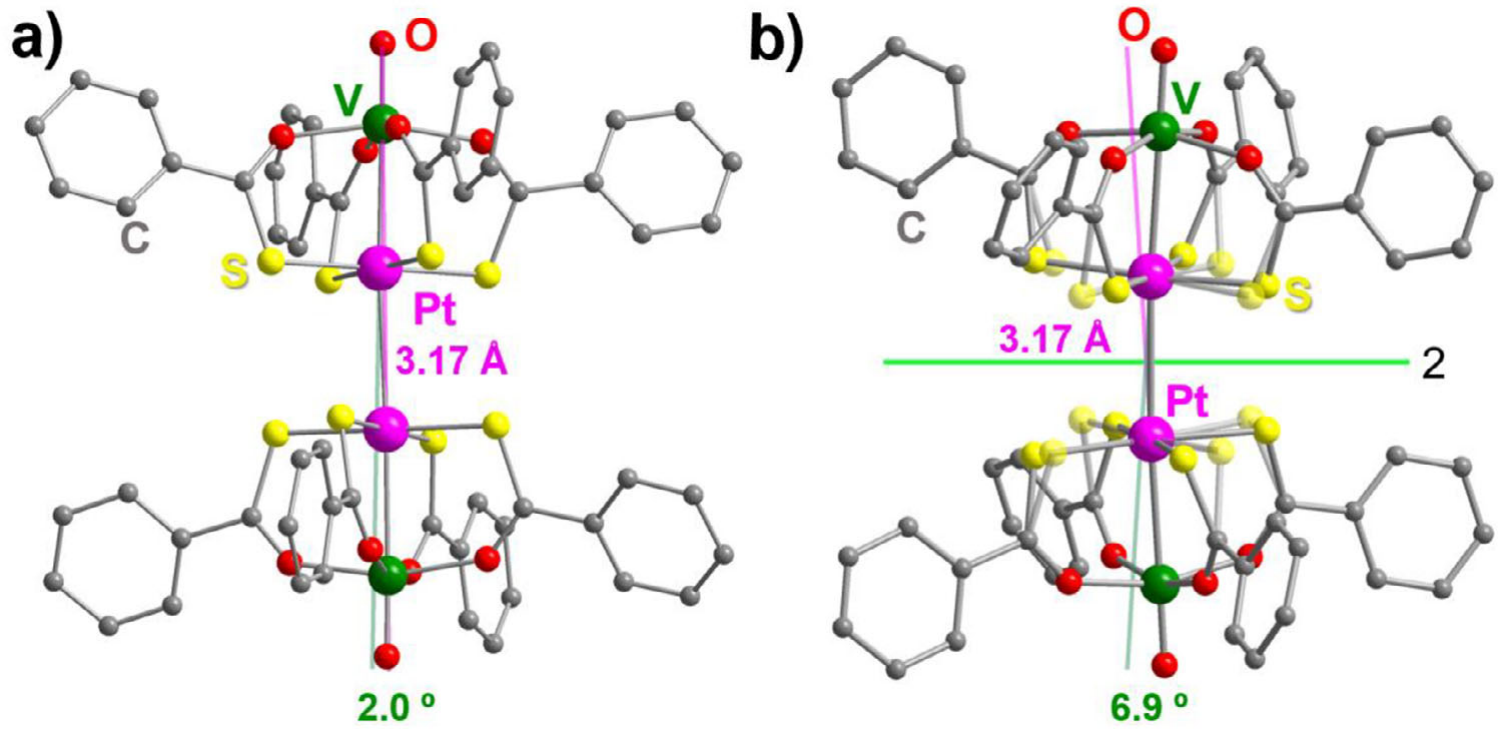
Structures of staggered dimers in (a) **1**·0.875tol (mol1⋅⋅⋅mol2) and (b) α‐**1**·0.5tol. Hydrogen atoms are omitted for clarity. In panel (b), the lower occupancy components of disordered S atoms are drawn in transparent style.

**FIGURE 5:**
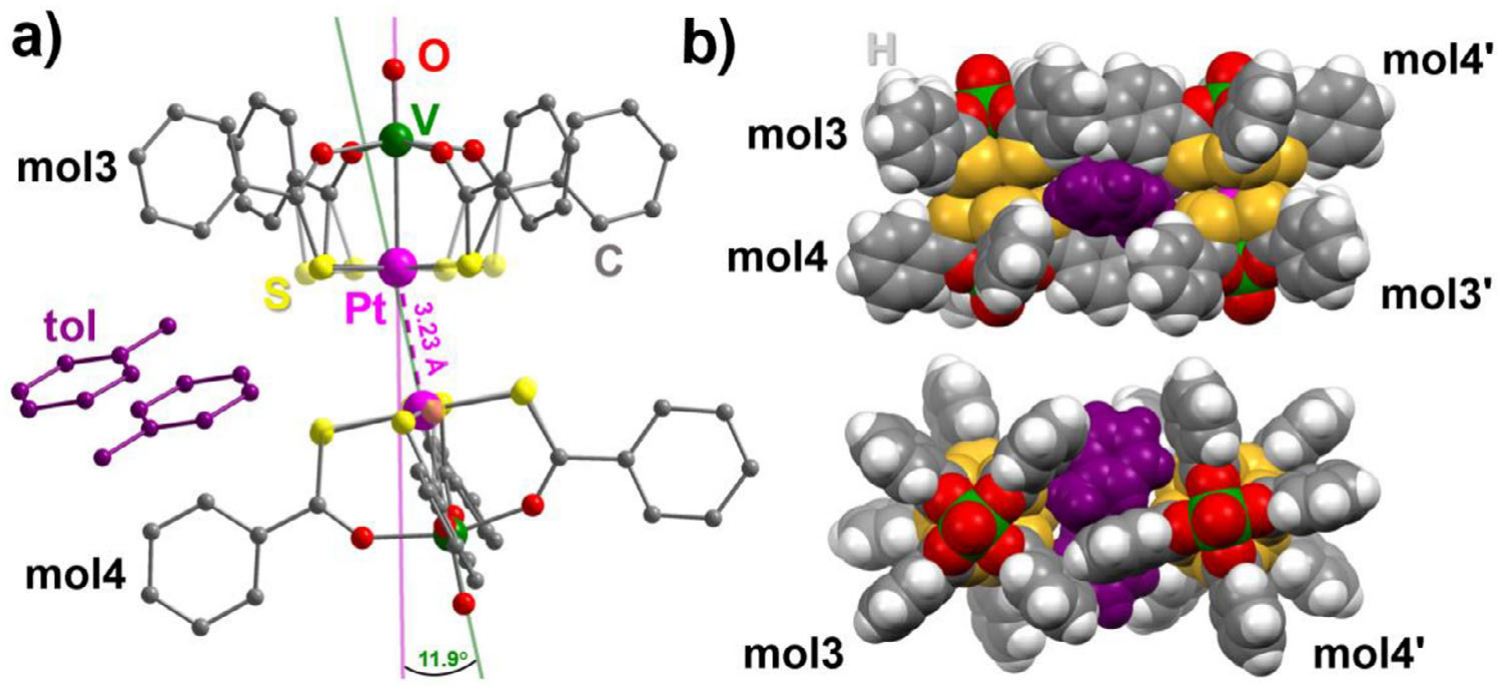
Structure of dimer mol3⋅⋅⋅mol4 in **1**·0.875tol: (a) ball‐and‐stick view, with hydrogen atoms omitted for clarity; (b) vdW spheres. In panel (a), the lower occupancy components of disordered S atoms are drawn in transparent style.

The general crystallographic features of the three new phases are as follows. Crystals of **1**·0.875tol belong to the triclinic space group *P* $\bar{1}$ and contain two crystallographically independent PW dimers in the asymmetric unit, both with a staggered configuration and *C*_1_ symmetry. One of the dimers (V1−Pt1⋅⋅⋅Pt2−V2 = mol1⋅⋅⋅mol2) is ordered within experimental resolution, and its structure is shown in Figure 4a. The second one (V3−Pt3⋅⋅⋅Pt4−V4 = mol3⋅⋅⋅mol4) exhibits resolvable rotational disorder along the molecular axis (Figure 5). According to powder X‐ray diffraction (PXRD), **1**·0.875tol is the dominant phase in crystallization batches (Figure S1).

**FIGURE 6:**
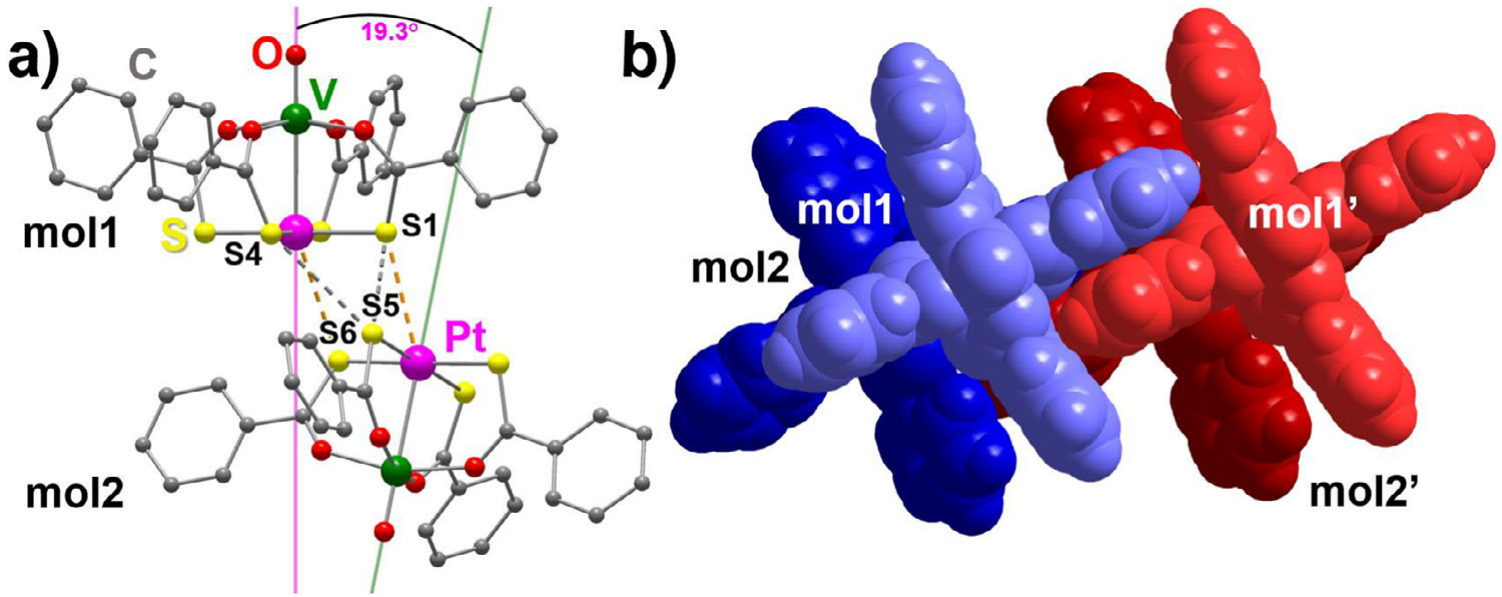
Structure of a dimer in β‐**1**·0.5tol: (a) ball‐and‐stick view, with hydrogen atoms omitted for clarity; (b) two neighboring dimers in vdW spheres, interacting via π‐stacking.

The first hemi‐toluene solvate, α‐**1**·0.5tol, crystallizes in the monoclinic space group *C*2/*c* and its asymmetric unit contains a single PW with rotationally disordered PtS_4_ moieties. Staggered dimers with *C*_2_ symmetry are then generated by twofold axes parallel to the unit‐cell *b* axis and approximately normal to the V−Pt direction (Figure 4b). The second hemi‐toluene solvate, β‐**1**·0.5tol, is triclinic (*P* $\bar{1}$ space group), and the asymmetric unit comprises a highly distorted dimer with *C*_1_ symmetry (Figure 6).

Comparing now the different molecular structures, all PWs in the series exhibit very similar geometrical parameters. The V−Pt distances span the range 2.78‐2.86 Å, with the staggered dimers showing the longest values, while the V═O bond length is rather insensitive to the dimerization motif. One interesting parameter is the intramolecular twisting angle τ_intra_, which takes on sizeable values (11.6‐20.6°) and implies that each PW has helical chirality. Curiously, the two neighboring molecules in all nondisordered toluene solvates and in **2** have the same handedness, while they necessarily have opposite handedness in the centrosymmetric dimers of **1**·CH_2_Cl_2_ and **1**·0.5hex. The values of τ_intra_ do not otherwise show an appreciable trend across the series.

On the other hand, differences emerge clearly when examining intermolecular descriptors like the Pt⋅⋅⋅Pt' and Pt⋅⋅⋅S' distances and the ϕ, ϕ', and γ angles defined in Figure 3a. Among the compounds listed in Table 1, the dimers in **1**·0.875tol, α‐**1**·0.5tol, and **2** display the shortest Pt⋅⋅⋅Pt’ contacts (3.17‐3.23 Å), the longest minimum Pt⋅⋅⋅S' separations (> 3.7 Å), and ϕ angles closest to 180° (170.4‐177.2°). These values are within the range observed for staggered dimers of Me‐ and Ph‐substituted PWs (see below). In particular, the smallest deviations from a linear metal arrangement are observed in mol1⋅⋅⋅mol2 of **1**·0.875tol and in **2**. In these complexes, the ϕ angles are straight within 4°, the collinearity angle γ does not exceed 2°, and the Pt⋅⋅⋅Pt' distances are identical within experimental error. To accommodate such short Pt⋅⋅⋅Pt' separations, the two PWs are rotated relative to one another. In dimers of coaxial or quasi‐coaxial PWs, this rotation can be quantified by averaging the smallest set of S−Pt···Pt'−S' dihedrals to give the intermolecular twisting angle τ_inter_ (Figure 3b). Table 1 shows that the value of τ_inter_ in mol1⋅⋅⋅mol2 of **1**·0.875tol (Figure 4a) is much closer to 45° (perfect staggering) than in the thioacetate analogue **2** (42.2 vs. 32.6°). However, DFT calculations indicate that isolated staggered dimers of thioacetate and thiobenzoate PWs should have similar configurations, suggesting that the different τ_inter_ values are likely to arise from crystal packing effects (vide infra).

The dimers in α‐**1**·0.5tol are virtually identical to those found in mol1⋅⋅⋅mol2 of **1**·0.875tol and in **2** in terms of Pt⋅⋅⋅Pt' distance and ϕ angle. However, γ reaches 6.890(16)°, resulting in a slightly bent arrangement (Figure 4b).

The second independent dimer in **1**·0.875tol (mol3⋅⋅⋅mol4) displays an even more distorted staggered configuration (Figure 5a), with a γ value reaching 11.87(4)°. This is presumably caused by the two toluene molecules sandwiched between neighboring dimers in the center of the unit cell (Figure 5b). The tilted configuration seems to be supported by weak T‐shaped π‐stacking interactions between Ph substituents within the assembly of two dimers and by short S⋅⋅⋅S' contacts at 3.18‐3.36 Å (affected by disorder), which fall below the sum of vdW radii (3.66 Å) [3]. We note that, according to the CSD [19], γ values larger than 5° are rarely encountered (Table S2) and that the γ angle found in mol3⋅⋅⋅mol4 is indeed similar to the largest value reported prior to this work, namely 11.90° in [PtNi(SOCPh)_4_(pyNO_2_)] [4]. In both cases, the noncollinearity seems to be supported by π‐stacking interactions between neighboring dimers and two short S···S' contacts within a dimer.

The structures of **1**·CH_2_Cl_2_, **1**·0.5hex, and β‐**1**·0.5tol are based on markedly noncoaxial dimers with much longer Pt⋅⋅⋅Pt' distances (3.84‐4.51 Å), Pt⋅⋅⋅S' contacts well below the sum of vdW radii (3.13‐3.38 vs. 3.55 Å) [3], and considerably bent V−Pt⋅⋅⋅Pt' arrangements (126.8‐143.6°). The relative orientation of the two PW units is, however, significantly different in the three solvatomorphs. Since the square dimers in **1**·CH_2_Cl_2_ and **1**·0.5hex are centrosymmetric, the two V−Pt vectors are strictly collinear (γ = 0), and the twisting angle τ_inter_ is exactly zero by symmetry. The S atom engaged in the shortest Pt⋅⋅⋅S' contact (3.13‐3.16 Å) is positioned virtually along the V−Pt direction of the neighboring molecule (V−Pt⋅⋅⋅S' = 173.7‐176.2°) [7, 18].

By contrast, the dimers in β‐**1**·0.5tol have no internal symmetry, and the two V−Pt vectors are markedly not collinear, with γ = 19.311(15)° (Figure 6a), the largest value ever reported for a PW dimer. In addition, the pair of reciprocating intermolecular Pt⋅⋅⋅S' contacts is also different with respect to those observed in **1**·CH_2_Cl_2_ and **1**·0.5hex. The S atoms are positioned off‐axis, with V−Pt⋅⋅⋅S' angles of 160.75(2) and 153.31(2)°, and the S atoms are ∼0.2 Å farther from the Pt atoms than in the square dimers. Tilting of the two PWs within the dimer is additionally supported by a pair of S⋅⋅⋅S' contacts at 3.347(1) (S1⋅⋅⋅S5) and 3.564(2) Å (S4⋅⋅⋅S5), hence less than the sum of vdW radii (3.66 Å) [3]. Presumably, such a strong distortion is imposed by the packing motif, as intermolecular π‐stacking interactions between phenyl rings assemble the dimers into rows parallel to the *a* axis (Figure 6b). At the same time, additional π‐stackings occur between molecular rows related by inversion (Figure 2), resulting in the highest crystal density among the three toluene solvates (at 200 K, 1.815 g cm^−3^ vs. 1.761 in **1**·0.875tol and 1.759 in α‐**1**·0.5tol).

### 2.3 DFT Calculations

Our structural data demonstrate that intermolecular interactions in crystals of **1** have an utmost flexibility, leading to staggered quasi‐coaxial, square, or more distorted dimeric assemblies depending on the exact crystalline phase. Notably, the three crystalline phases of toluene solvates delineate an even more complex situation than that reported by Larsen *et al.* for the staggered and eclipsed forms of [PtNi(SOCMe)_4_(pyNO_2_)] [4]. Therefore, it is of interest to estimate the interaction electronic energy in the gas‐phase dimers as a function of dimerization mode. Dahl *et al*. reported that staggered dimers of [PtCo(SOCPh)_4_(H_2_O)] and [PtNi(SOCPh)_4_(H_2_O)] are more stable than the separated monomer pairs by ca. 20 kcal mol^−1^ [13]. Larsen *et al.* computed a comparable stabilization energy in eclipsed dimers of [PtNi(SOCMe)_4_(pyNO_2_)] [6], whereas Sandoval‐Olivares and coworkers arrived at significantly larger interaction energies in dimers of [PtCo(SOCMe)_4_(H_2_O)] and [PtNi(SOCMe)_4_(H_2_O)] [20].

Starting from the crystallographic coordinates of **1**·CH_2_Cl_2_, **1**·0.875tol (mol1⋅⋅⋅mol2), and **2**, we used two geometry optimization strategies: optimization of H atom positions only and full geometry optimization (the structure found in the dichloromethane solvate was chosen as representative of both square dimers). In each case, we evaluated the interaction electronic energy as Δ*E*_int_ = *E*_d_ − 2*E*_m_, where *E*_d_ and *E*_m_ are the electronic energy values of the dimer and of each corresponding separated monomer, respectively, in the gas phase (as usual, a negative Δ*E*_int_ value indicates that the dimer is more stable than the pair of separated monomers) [4, 6, 13]. Calculations were done by the DFT method implemented in ORCA 5.0.4 using the PBE0 functional, the def2‐TZVPP basis set, and the D3BJ dispersion correction [4, 5]. The results are presented in Table 2. The value of −Δ*E*_int_ spans a remarkably narrow interval (14.8‐18.3 and 15.6‐18.6 kcal mol^−1^ for H‐only and full geometry optimizations, respectively). Comparison between the optimized staggered and square dimers of thiobenzoate **1** shows that the former is more stable by 2.8 kcal mol^−1^ (H‐only) or 2.0 kcal mol^−1^ (full), suggesting a shallow energy profile. Worth noting is also that Δ*E*_int_ in the formation of staggered coaxial dimers is significantly influenced by the R substituent on the thiocarboxylate ligands, being 3.0‐3.5 kcal mol^−1^ more favorable with R = Ph than with R = Me. The difference is ascribed to dispersion interactions, which are almost entirely responsible for dimer formation (Table 2) and are 2.9‐3.7 kcal mol^−1^ stronger in the thiobenzoato staggered dimers, presumably because of long‐range ligand⋅⋅⋅ligand contributions [13]. We also checked the dependence of Δ*E*_int_ on the relative handedness of the two monomers in staggered coaxial dimers of **2**. Full geometry optimization after reversing the handedness of one of the two PWs indicated that a heterochiral dimer would be only marginally more stable than the homochiral dimer found in the crystal structure (−Δ*E*_int_ = 16.0 vs. 15.6 kcal mol^−1^).

**TABLE 2:** Interaction electronic energy (kcal mol^−1^), dispersion contribution to interaction electronic energy (kcal mol^−1^), and selected interatomic distances (Å) and angles (°) in PW dimers after geometry optimization at the PBE0/def2‐TZVPP/D3BJ level (gas‐phase).^a^

Compound	−Δ*E*_int_	−Δ*E*_int_^disp^	V─Pt, V'─Pt'	Pt···Pt'	Pt···S'^b^	ϕ, ϕ'^c^	τ_inter_ ^d^
**1**·CH_2_Cl_2_	(15.5) 16.6	(15.8) 17.2	2.866, 2.866	3.517	3.468	152.65, 152.66	0.0
**1**·0.875tol^e^	(18.3) 18.6	(17.1) 17.9	2.867, 2.868	3.118	3.886	179.65, 179.03	41.4
**2**	(14.8) 15.6	(14.2) 14.2	2.922, 2.922	3.159	3.924	179.28, 179.28	40.7

^a^ Values of −Δ*E*_int_ and −Δ*E*_int_^disp^ in parentheses were obtained by optimizing H positions only; in this case, geometrical parameters are the same as those in Table 1; all remaining data are from full geometry optimizations.

^b^ Shortest Pt···S' contact.

^c^ See Figure 3a.

^d^ See Note d in Table 1 and Figures 3b‐c.

^e^ Dimer of mol1···mol2.

According to the thermochemical data of the fully optimized structures, adding zero‐point energies, thermal vibrational corrections, and electronic and vibrational entropy terms to electronic energies makes dimerization at 298.15 K even more exergonic by 3.9‐5.3 kcal mol^−1^, primarily because of the favorable change of vibrational entropy. However, the trends in Table 2 remain unvaried, with the staggered thiobenzoate dimer more stable than the “square” dimer by 1.6 kcal mol^−1^, and the formation of the staggered dimer ca. 4.0 kcal mol^−1^ more exergonic with thiobenzoate than with thioacetate ligands. Gibbs free energy changes upon dimerization (Δ*G*_int_) at 298.15 K and 1 atm amount to less than 2.5 kcal mol^−1^ in absolute value because of the unfavorable rotational and translational entropy changes. The inclusion of implicit solvation by toluene, evaluated using the conductor‐like polarizable continuum model (CPCM), leads to only marginal changes in these Δ*E*_int_ and Δ*G*_int_ values.

In the fully optimized structures of staggered dimers, that is, **1**·0.875tol (mol1⋅⋅⋅mol2) and **2**, the metal array is linear within 1° or less, and deviations from perfect staggering do not exceed 5° (Table 2). The effect of the R substituent on τ_inter_ is here marginal, both R = Me and Ph yielding intermolecular twisting angles similar to that observed in mol1⋅⋅⋅mol2 of **1**·0.875tol (Table 1).

**FIGURE 7:**
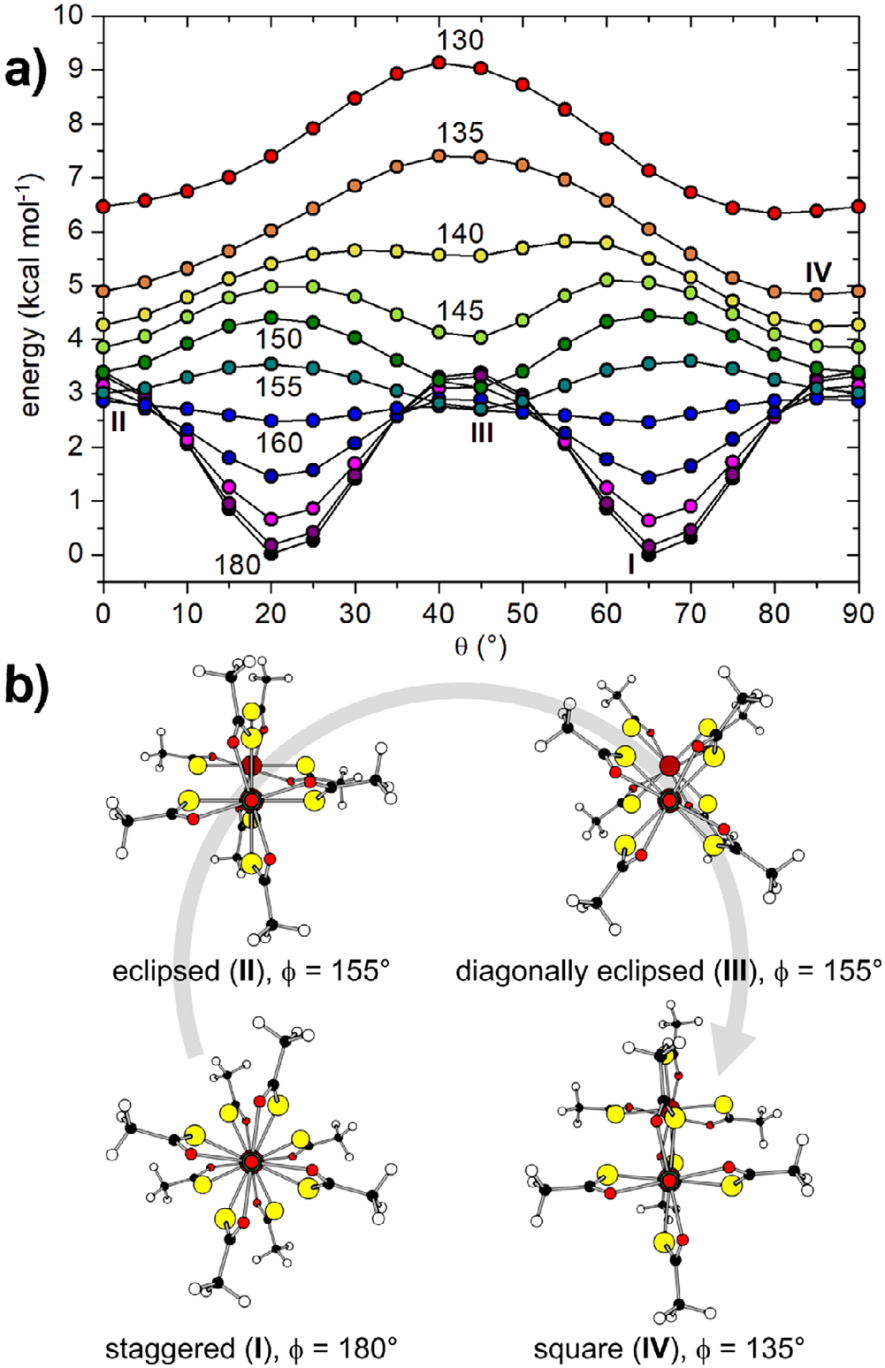
(a) θ‐ and ϕ‐dependent electronic energy profiles of PW dimers in the gas phase, calculated by relaxed surface scans using 5° angular steps. Curve labels are ϕ values in degrees. The electronic energy of the lowest‐energy structure is set to zero. (b) Models corresponding to the (local) energy minima at ϕ = 180° (**I**), 155° (**II** and **III**), and 135° (**IV**) in panel (a).

Full optimization of **1**·CH_2_Cl_2_, however, yields a significantly shorter Pt···Pt' separation than in the crystal (3.52 vs. 3.84 Å), with wider ϕ angles (∼153 vs. ∼144°) and a Pt···S' contact ca. 0.34 Å longer than experimentally observed (3.47 vs. 3.13 Å). The reason is that the fully optimized dimer has γ and τ_inter_ equal to zero, like the starting structure, but does not show a square configuration. In fact, the angles θ and θ' in Figure 3c (±36.7°) are much wider than in the X‐ray structure (±4.9°), meaning that the PtS_4_ moieties are approximately bisected by the metal plane rather than having one Pt−S bond roughly in that plane. The resulting geometry is indeed similar to structure **III** in Figure 7b (see below).

The geometry dependence of the interaction electronic energy in thioacetate **2** was further investigated at the same level of theory by relaxed surface scans, that is, fixing one or more geometrical parameters while relaxing all remaining degrees of freedom. We recall that, in the vast majority of PW dimers, the two Tr−M vectors are approximately or exactly collinear (γ < 5°), which implies virtually coplanar metal ions and ϕ ∼ ϕ' (see below). For instance, among the complexes gathered in Table 1, the staggered dimers in **1**·0.875tol (mol1⋅⋅⋅mol2) and **2**, as well as the centrosymmetric square dimers of **1**·CH_2_Cl_2_ and **1**·0.5hex, meet this criterion. The structure of a single PW was first pre‐optimized with imposed *C*_4_ symmetry and then used as a rigid fragment to assemble pairs of collinear PWs with γ = 0 (Figures 3b,c). The scanned geometrical parameters were the displacement of one PW relative to the other (ϕ) and the orientation of the PWs vs. the metal plane (θ, θ’). It is noted that we assumed θ = θ' for simplicity and varied θ from 0 to 90°, because the two PWs have the same handedness and the energy profiles are only approximately mirror symmetric about θ = 45°. With these constraints, only the Pt⋅⋅⋅Pt’ distance was treated as an adjustable parameter, yielding the electronic energy values plotted in Figure 7a and the selected geometrical parameters graphically presented in Figure S4.

For ϕ ∼ 180‐160° (i.e., for coaxial or quasi‐coaxial PWs), the minimum energy structures correspond to the two staggered and quasi‐degenerate configurations at τ_inter_ = 2θ ∼ 45 and 135° (**I**). On the other hand, the eclipsed configurations (τ_inter_ ∼ 0 and 90°) are local energy maxima. As ϕ is progressively reduced, the energy of the staggered structures increases until at ca. 155° the eclipsed configurations become local minima. It is relevant that significant deviations from coaxiality afford two distinct eclipsed structures with virtually the same energy. The first structure (**II**) has θ ∼ 0, implying that both PWs position one of their Pt−S bonds on the metal plane. The second one (**III**), here denoted as *diagonally eclipsed*, has both PtS_4_ moieties twisted away from the metal plane by θ ∼ 45° and is similar to that reached by full optimization of **1**·CH_2_Cl_2_ (ϕ ∼ 153°). Therefore, at these ϕ values the two PWs can be rotated away from the metal plane without a significant energy penalty with both R substituents. From ϕ = 155 to 145°, the energy difference between the two structures remains well below 0.5 kcal mol^−1^; larger displacements progressively favor an eclipsed configuration with θ ∼ 0, which eventually becomes the only energy minimum at ϕ = 135° (**IV**) or below.

Note that the two quasi‐degenerate staggered configurations with ϕ = 180° are the most stable structures. Furthermore, the (local) minima in each curve become invariably less stable as ϕ decreases, and the difference from **I** reaches ca. 6 kcal mol^−1^ at ϕ = 130°. Across the ϕ‐scan, the Pt⋅⋅⋅Pt' separation at these (local) minima increases regularly from 3.17‐3.18 to 4.59 Å (Figure S4). The ϕ‐dependence of the shortest Pt⋅⋅⋅S' contact is more complex, since this geometrical parameter is severely influenced by the value of θ. In the staggered dimers, Pt⋅⋅⋅S' decreases from 3.94‐3.95 to 3.48‐3.52 Å as ϕ is reduced from 180 to 160°. For smaller ϕ angles, the local energy minima at θ ∼ 0 and 45° imply Pt⋅⋅⋅S' distances differing by 0.3 Å or more, with the closest Pt⋅⋅⋅S' contacts (3.13 Å) occurring in square dimers (Figure S4).

### 2.4 Statistical Analysis

With this knowledge in hand, we turned to statistical data, relying mostly on CSD (CCDC 2025.2.0) [19]. In the database, we found 62 entries (with available 3D coordinates) containing pairs of [MTr(SOCR)_4_L] molecules with M···M' and M···S' contacts not exceeding 5 Å in length, which we herein consider indicative of dimer formation (M = Pt, Pd; Tr = first‐row transition metal; R = any; L = Tr‐coordinated axial ligand). Matching the above metrical criterion are also the structures of **1**·0.5hex [18] and of three unpublished Pd derivatives from our group [8], affording a total of 66 entries. Since each structure can contain inequivalent dimers and the PWs within each dimer can themselves be inequivalent, there are 89 crystallographically unique [MTr(SOCR)_4_L] units in the dataset (Table S2). The array was further simplified by only considering complexes with R = Me or Ph and avoiding data redundancy from temperature‐dependent studies, yielding a final set of 59 (R = Me) and 16 (R = Ph) units, engaged in the formation of 45 (R = Me) and 14 (R = Ph) dimers. The toluene solvates of **1** reported in this work also match our metrical criterion and provide 4 additional thiobenzoate dimers comprising 7 crystallographically independent PWs.

The excluded entries show that coordination of bulky axial ligands to a transition metal causes, in some cases, crystallization in monomeric form. Moreover, bulky solvent molecules, such as acridine in the complexes [PtTr(SOCMe)_4_(H_2_O)]·acridine (Tr = Co [21], Zn [22]), also prevent dimerization. However, these structures invariably contain other types of short stabilizing contacts. Examples are the weak S···H interactions in complexes with terminal quinuclidine ligands [23], the hydrogen bonds between the Me substituent of thioacetate ligands and the cyano‐group of 4‐cyanopyridine [2], the π‐stacking interaction between the Ph group of thiobenzoate and the aryl system of the L ligand in [PtTr(SOCPh)_4_(pySMe)] (Tr = Co, Ni, Zn; pySMe = 4‐(methylsulfanyl)pyridine) [24], and the strong π‐stacking interactions between acridine molecules, which, presumably, are more energetically favorable than PW dimerization.

**FIGURE 8:**
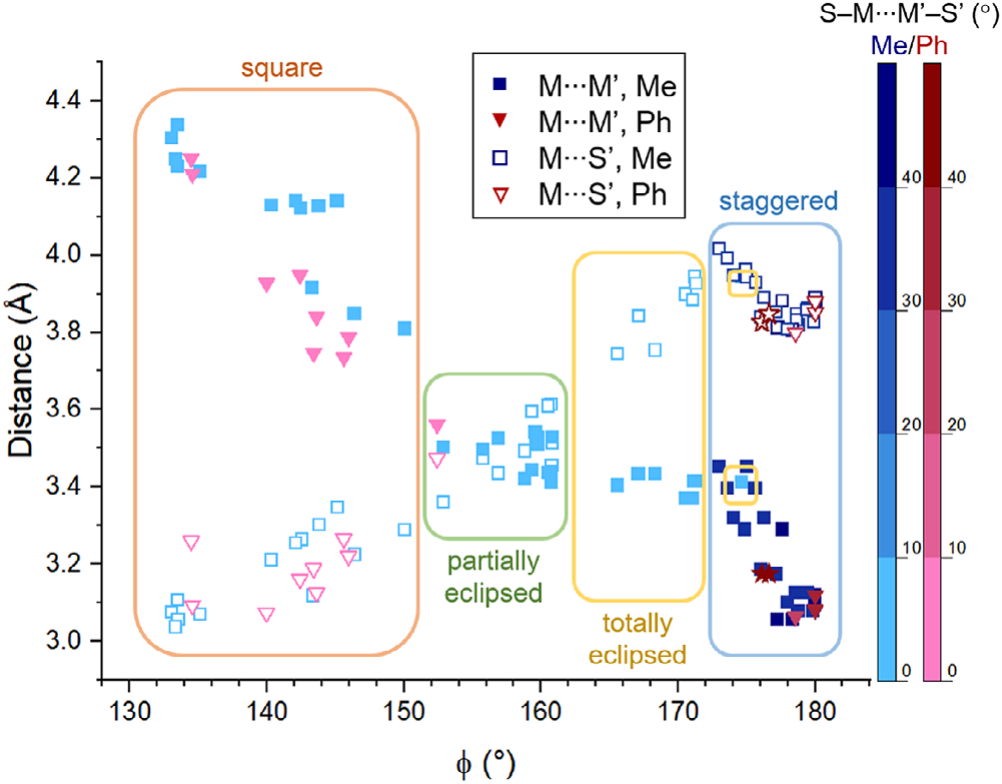
Statistical distribution of M···M' and M···S' contact distances plotted against the ϕ angle. The colors encode the absolute value of the minimal torsion angle S–M···M'–S' in the corresponding fragment. Only collinear structures with γ < 5° are displayed. Data according to CCDC 2025.2.0 [19] and other sources. Stars represent the data for **1**·0.875tol (mol1···mol2).

As mentioned in Section 2.3, the vast majority of known PW dimers have collinear or quasi‐collinear Tr−M vectors within 5° (Figures 3b, c) and match the model used for DFT calculations. More precisely, only 8 of 63 dimers (including those newly reported in this work) have γ > 5° (Tables 1 and S2). Therefore, we only focused on dimers with γ < 5° and described their structures using four geometrical parameters for each fragment: the Tr─M···M' angle (ϕ), the M···M' and M···S' distances, and the minimal S–M···M'–S' torsion angle, which qualitatively describes the relative twisting of the two PWs (Table S2). Figure 8 presents M···M' and M···S' vs. ϕ plots, distinguishing between thioacetate and thiobenzoate dimers and encoding the torsion angle information in the symbol color.

The graph clearly highlights the family of *staggered* dimers, which cluster at ϕ = 180‐173° while displaying the shortest M···M' separations (3.1‐3.4 Å) and long M···S' contacts (3.8‐4.0 Å). The lower angular boundary for staggered dimers is surprisingly sharp, as clearly shown by the abrupt decrease of the twisting angle below ϕ = 173°. The available data contain only one exception: compound [{Na(12‐crown‐4)_2_}{PtCo(SOCMe)_4_(NCS)}]·acetone [10] has ϕ = 174.64° but a virtually zero twisting angle and thus belongs to the family of eclipsed dimers. Further inspection of the cluster of staggered dimers reveals that the M···M' and M···S' distances both increase quite significantly (0.2‐0.4 Å) as ϕ decreases from 180 to 173°. Below ϕ = 173°, M···M' increases much slower, finally reaching 4.3 Å at 133°, while M···S' decreases with approximately the same slope, approaching ca. 3.0 Å as the Tr─M···M' angle reaches its lower limit. The two plots intersect near the middle of the graph, at ϕ ∼ 160°, a value that marks the transition from totally eclipsed dimers (M···M' < M···S') to partially eclipsed dimers (M···M' > M···S') in Doerrer's classification (Figure 1). All reported dimers with ϕ in the range from 173 to 165° indeed have M···M' < M···S' by more than 0.3 Å and thus meet the definition of *totally eclipsed* species. The cluster of dimeric structures with ϕ = 161‐158° shows no clear trend as a function of ϕ; expectedly, this parameter is of limited utility close to the crossing point. From just below the crossing point (ϕ < 157°) to the lower ϕ limit available, all data indicate M···S' as the shortest intermolecular contact, without known exceptions. In this angular range, the distinction between *partially eclipsed* and *square* dimers is somewhat arbitrary, being based on the value of ϕ. From the graph, it seems quite sensible to set the boundary at ϕ ∼ 152° (M···M' ∼ 3.56 Å), because the next‐lower ϕ value leads to an abrupt increase of M···M' to ∼3.7‐3.8 Å, exceeding M···S' distance by more than 0.5 Å. Figure S5 shows the position of the bent dimers relative to the general trend.

Although the complexes herein considered comprise mostly thioacetate derivatives, with thiobenzoate dimers amounting to only 28% of the total, the two families follow similar trends in Figure 8, except for the fact that thiobenzoate dimers cluster above ϕ = 176° (staggered dimers) and below 152° (square dimers). Besides this, neither the M···M' distance nor the dimerization mode is affected by the nature of the first‐row transition metal (Figure S6).

**FIGURE 9:**
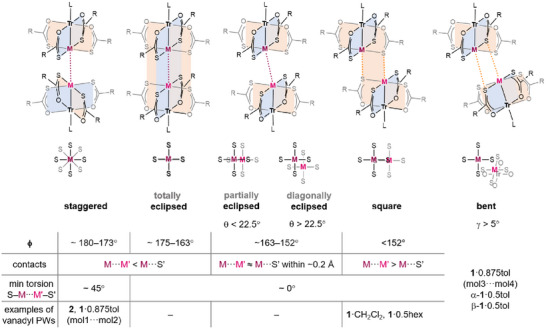
Expanded classification of PW dimers. For all classes except bent dimers, γ < 5°.

Based on the above‐described statistical analysis, the classification scheme proposed by Doerrer *et al.* was refined as depicted in Figure 9. By limiting the classification to collinear or quasi‐collinear PW dimers (γ < 5°), we have shown that the four classes of staggered, totally eclipsed, partially eclipsed, and square dimers occur in distinct ϕ ranges. Inspired by the results of DFT calculations, we also examined the orientation of the MS_4_ moieties with respect to the metal plane. As a descriptor, we chose the minimal dihedral angle between (TrMS) and (TrMM'Tr') planes. Considering the approximate fourfold symmetry of the MS_4_ moieties, this angle has 45° as an upper value and corresponds to θ in Figure 3c. When plotted against ϕ, the θ angle spans rather evenly the whole range from 0 to 45° (Figure S7). However, large θ values lead to significantly different geometries only for strongly noncoaxial PWs, as those found in partially eclipsed dimers. Figure S7 shows that the θ values of many partially eclipsed dimers cluster in the upper half of the accessible interval (θ > 45/2 = 22.5°). To account for the largely different geometries adopted by partially eclipsed dimers as a function of θ, we expand the classification scheme of Doerrer *et al.* by introducing the new subcategory of *diagonally eclipsed* structures, which comprises the group of dimers, previously classified as partially eclipsed, with θ > 22.5°.

## 3 Conclusion

The heterobimetallic PW complex [PtVO(SOCPh)_4_] (**1**) undergoes dimerization in the crystalline state to afford a large variety of structures supported by metallophilic (Pt⋅⋅⋅Pt') or Pt⋅⋅⋅S' interactions. Three crystalline toluene solvates were identified, namely **1**·0.875tol, α‐**1**·0.5tol, and β‐**1**·0.5tol, which contain dimeric assemblies ranging from staggered quasi‐coaxial to heavily bent noncoaxial dimers. Although gas‐phase DFT calculations indicate staggered coaxial dimers as the lowest‐energy structural motif, the potential energy surface for dimerization is shallow, and alternative geometries are accessible within a few kcal mol^−1^. We conclude that interdimer forces or interaction with lattice solvent molecules are likely to play a pivotal role in determining the exact structure adopted in the crystalline state.

The structures of [MTr(SOCR)_4_L] compounds reported in the CSD show that, in the vast majority of cases, dimerized PWs have collinear Tr−M vectors within 5°. In such cases, the dependence of the M···M' and M···S' distances on the Tr−M⋅⋅⋅M' angle (ϕ), along with the value of the smallest S–M···M'–S' dihedral, clearly indicates transitions between staggered, totally eclipsed, partially eclipsed, and square configurations, according to the classification introduced by Doerrer *et al.* [1, 2]. Therefore, ϕ is a key parameter in determining the internal structure adopted by the dimer. Further analysis of the PW twisting relative to the metal plane revealed that most partially eclipsed dimers belong to the subcategory of *diagonally eclipsed* structures, in which both PWs are significantly twisted away from the metal plane. Relaxed surface scans by DFT clearly displayed the transition from staggered to square dimers through eclipsed configurations as ϕ is progressively reduced from 180° and confirmed that diagonally eclipsed structures are local minima in the potential energy surface.

Dimers in which deviations from collinearity exceed 5° are here denoted as *bent* dimers. Examples are found both in the literature and in the new solvatomorphs of **1** described in this work. In particular, β‐**1**·0.5tol features the most noncollinear PW pair so far reported within the pool of [MTr(SOCR)_4_L] compounds. Bent dimers are encountered throughout the ϕ range, and therefore all other parameters can vary in representatives of this family.

Dimerization through M···M' or M···S' contacts introduces antiferromagnetic coupling between vanadyl ions, with singlet‐triplet gaps reaching several wavenumbers in staggered dimers [5, 7, 8]. This tendency, which profoundly affects the magnetic behavior of the individual PWs, must be taken into proper consideration when planning to use vanadyl PWs as isolated spin qubits or as components of scalable qubit arrays. As mentioned above, dimers dissociate into monomers in organic solutions, which provides the simplest route to access magnetically diluted qubits [5, 8]. In a more application‐oriented strategy, crystals of an isostructural but diamagnetic analogue (typically, the corresponding titanyl or zinc compound) [25] can be doped with vanadyl ions. When applied to dimeric PWs, this approach is expected to yield dimers containing at most one paramagnetic center, thus ensuring suppression of through‐bond coupling mediated by M···M' or M···S' contacts.

Our work shows, however, that vanadyl PWs are easily displaced from the most stable, staggered dimeric arrangement by competing intermolecular forces, like those responsible for crystal cohesion. Doerrer *et al.* indeed used **1** and **2** as metalloligands to build heterotrimetallic complexes with lanthanide ions and found no evidence of Pt···Pt' or Pt···S' interactions in their crystal structures [7]. Therefore, the potential of vanadyl PWs in molecule‐based quantum technologies may not be severely limited by their tendency to form dimers through M···M' or M···S' contacts.

## 4 Experimental Section

### 4.1 General Methods and Synthesis

Toluene, *n*‐hexane, and CDCl_3_ (99.8 atom % D) were stored over activated 4 Å molecular sieves prior to use. Elemental analysis (CHN) was performed using a ThermoFisher Scientific Flash 2000 analyzer. ^1^H NMR spectrum was recorded at 298 K on a ∼0.01 M solution in CDCl_3_ using an Avance 400 spectrometer from Bruker Biospin. Chemical shifts (δ) are expressed downfield from TMS and referenced to the residual proton resonances of the solvent (δ = 7.26 ppm) [26].

The complex **1** was synthesized by a modified literature procedure [5, 7]. NaSOCPh was isolated in solid form, and the reaction time between NaSOCPh and K_2_PtCl_4_ was reduced to 1 h. This procedure yielded the raw material in 78% yield in the form of a green powder on a glass filter. The compound was collected from the filter with hot toluene (ca. 60–80°C) and introduced in a vial (ca. 15 mL of toluene were required to dissolve 79 mg of compound). The solution was concentrated in vacuum to 1.5 mL, giving a fine crystalline suspension, then the tightly closed vial was placed in an inclined position in an oven at 60°C for four days. In the course of this time, grassy‐green crystals formed, and they were structurally characterized by SXRD. The mother liquor was decanted, crystals washed with *n*‐hexane (5 × 1 mL), and dried in a vacuum. According to elemental analysis and ^1^H NMR, the composition of the product is **1**·0.3tol·0.1hex (69% total yield referred to Pt). ^1^H NMR (Figure S2, 400.13 MHz, CDCl_3_): δ = 8.48 (br s, 4H; *p*‐C_6_ *H*_5_), 4.32 ppm (br s, ∼8H; *m*‐C_6_ *H*_5_); elemental analysis calcd (%) for **1**·0.3tol·0.1hex (C_30.7_H_23.8_O_5_PtS_4_V, 847.00): C 43.53, H 2.83; found: C 43.64, H 2.76. Depending on washing and drying conditions, the toluene content per PW can range from 0.3 to 0.8; solvent‐free samples were never isolated.

### 4.2 X‐ray Crystallography

SXRD data were collected at 200 K on a Bruker‐Nonius X8APEX diffractometer, equipped with a Mo‐Kα generator, an area detector, and a Kryoflex cryostat. One of the components of an epoxy resin was used for crystal handling, selection, and mounting on a MiTeGen Microloop or on the flame‐sealed tip of a glass capillary without significant loss of any lattice solvent. Matrix frames and data collection were done with APEX2 v1.0‐22 software, while data reduction was performed with SAINT v7.06A program and was followed by scaling and multi‐scan absorption correction using SADABS v2.10 [27]. The structures were solved by direct methods (SIR92 [28]) and refined by full matrix least‐squares methods on *F*_o_^2^ using SHELXL‐2018/3 program [29] and WINGX v2020.2 suite [30]. All nonhydrogen atoms were treated anisotropically, unless otherwise noted. H atoms were assigned isotropic displacement parameters *U* = 1.5*U*_eq_(C) for methyl groups and *U* = 1.2*U*_eq_(C) for the other H atoms. When necessary, restrained anisotropic refinement or isotropic treatment was used for solvent molecules and disordered S atoms. In some cases, the former were refined with geometrical restraints or constraints.

In the structure of **1**·0.875tol, slant‐plane Fourier maps taken in the mean planes of the PtS_4_ moieties (Figure S3) clearly show rotational disorder of the Pt coordination spheres. The two disordered components around Pt3 have markedly unequal occupations, whereas the S atoms around Pt4 are split into two positions with comparable occupations. Displacement ellipsoids of thiobenzoate O atoms are also elongated tangentially to the O−V bond, but we limited our modelling of disorder to the S atoms only. Refinement resulted in 0.637(15):0.363(15) and 0.508(19):0.492(19) occupation factors, respectively, indicating partially uncorrelated disorder.

In the structure of α‐**1**·0.5tol, the coordination environment of Pt1 also shows rotational disorder, with a refined ratio of 0.470(5):0.530(5) for the two components, suggesting a highly correlated disorder.

PXRD was carried out at room temperature (298 K) with a Malvern‐Panalytical Empirean MultiCore X‐ray powder diffractometer, equipped with an Empirean tube Cu LFF High Resolution (wavelength, λ = 1.54056 Å) at 40 kV and 40 mA, and with a capillary spinner sample stage rotating during the measurement (to reduce the impact of preferred orientation of the crystallites). The sample for PXRD analysis was prepared using freshly crystallized material stored under the mother liquor. Crystals were ground under the solution and filled into a Kapton capillary, which was then sealed with epoxy glue, following the method of Von Dreele [31]. The crystalline material in the capillary remained stable on a half‐year timescale. Data collection was performed in the range 2θ = 3–50°, in steps of 0.013° at a rate of 250 s per step. Powder patterns were simulated with Mercury 2024.2.0 [32] using a full‐width‐at‐half‐maximum of 0.1° in 2θ.

Deposition Numbers 2473728, 2473729, and 2473730 contain the supplementary crystallographic data for this paper. These data are provided free of charge by the joint Cambridge Crystallographic Data Centre and Fachinformationszentrum Karlsruhe Access Structures service.

### 4.3 DFT Calculations

DFT calculations were done using ORCA 5.0.4 [33, 34, 35, 36] with either the composite DFT approach B97‐3c [37, 38], which embeds the atom‐pairwise dispersion correction with the Becke‐Johnson damping scheme (D3BJ) [39, 40], or the PBE0 hybrid functional with def2‐TZVPP basis set [41] for all atoms and D3BJ dispersion correction [39, 40], as in previous work [4, 5]. Doublet and triplet spin states were assumed for all monomeric and dimeric structures, respectively. For platinum, we used an effective core potential to describe the inner core electrons [42]. DEFGRID3, TightSCF, and TightOPT settings were used throughout. Gas‐phase geometry optimizations (either H‐only or full) were done at the PBE0/def2‐TZVPP/D3BJ level and started directly from X‐ray coordinates. Full geometry optimizations were also carried out at the same level of theory with the inclusion of implicit solvation by toluene (CPCM [43]). Analytical calculation of vibrational frequencies in the harmonic approximation was carried out on fully optimized structures to check that a local energy minimum had been reached. Thermochemical data at 298.15 K and 1 atm were calculated using standard statistical mechanics equations for an ideal gas and assuming strictly harmonic vibrations.

For relaxed surface scans in the gas phase, the structure of a single PW in **2** was preliminarily fully optimized at the PBE0/def2‐TZVPP/D3BJ level with imposed *C*_4_ symmetry. It was subsequently used to build dimeric models consisting of pairs of collinear PWs (γ = 0). Collinearity was imposed by constraining ϕ and ϕ' to be equal and fixing the V−Pt⋅⋅⋅Pt’−V' dihedral to 180° (except for ϕ = 180°). ϕ and θ (= θ') were allowed to vary from 180 to 130° and from 0 to 90°, respectively, in steps of 5°. The initial Pt⋅⋅⋅Pt’ distance was that resulting from a rigid lateral displacement of one PW relative to the other, starting from 3.159 Å at ϕ = 180°. These 209 models were pre‐optimized at the B97‐3c level, treating the individual PWs as rigid fragments while constraining γ, ϕ, and θ to their initial values. This left the Pt⋅⋅⋅Pt' distance as the only adjustable parameter. The same constraints were applied in the final geometry optimization at the PBE0/def2‐TZVPP/D3BJ level.

## Author Contributions

Olga Mironova: synthesis & investigation, writing – original draft; Giacomo Bellini: synthesis & investigation; Alessio Nicolini: PXRD characterization, formal analysis, methodology; Andrea Cornia: crystallography, conceptualization, funding acquisition, project administration, methodology, DFT calculations, writing – original draft. All authors contributed to writing – review & editing, and writing relevant sections of the article.

## Conflicts of Interest

The authors declare no competing financial interest.

## Supporting information



PXRD pattern, ^1^H NMR spectrum, additional figures and tables on crystallographic analysis, DFT calculations, and statistics. Cartesian coordinates and thermochemical data of DFT optimized structures. CCDC 2473728, 2473729, and 2473730.
**Supporting File 1**: chem70500‐sup‐0001‐SuppMat.pdf.

**Supporting File 2**: chem70500‐sup‐0002‐DataFile.zip.

## Data Availability

The data that support the findings of this study are available in the supplementary material of this article.
