# Supplementary material for: Multiple Dimerization Modes in Thiocarboxylate Paddlewheel Complexes: A Comprehensive View of Energy Landscapes from DFT Calculations and Statistics
Source: Chemistry. 2025 Dec 2;32(15):e03004. doi: 10.1002/chem.202503004 (PMC13107499; doi:10.1002/chem.202503004)
Supplement: Supplementary file 1 — PXRD pattern, 1H NMR spectrum, additional figures and tables on crystallographic analysis, DFT calculations, and statistics. Cartesian coordinates and thermochemical data of DFT optimized structures. CCDC 2473728, 2473729, and 2473730. Supporting File 1: chem70500‐sup‐0001‐SuppMat.pdf. [file CHEM-32-e03004-s001.pdf]

Multiple Dimerization Modes in Thiocarboxylate Paddlewheel Complexes: a Comprehensive View of Energy Landscapes from DFT Calculations and Statistics

Olga Mironova, Giacomo Bellini, Alessio Nicolini, and Andrea Cornia*

Supporting Information

Table of contents

1. Synthesis S2
2. X-ray crystallography S3
3. DFT calculations S4
4. Statistical analysis S5
5. References S10

1. Synthesis

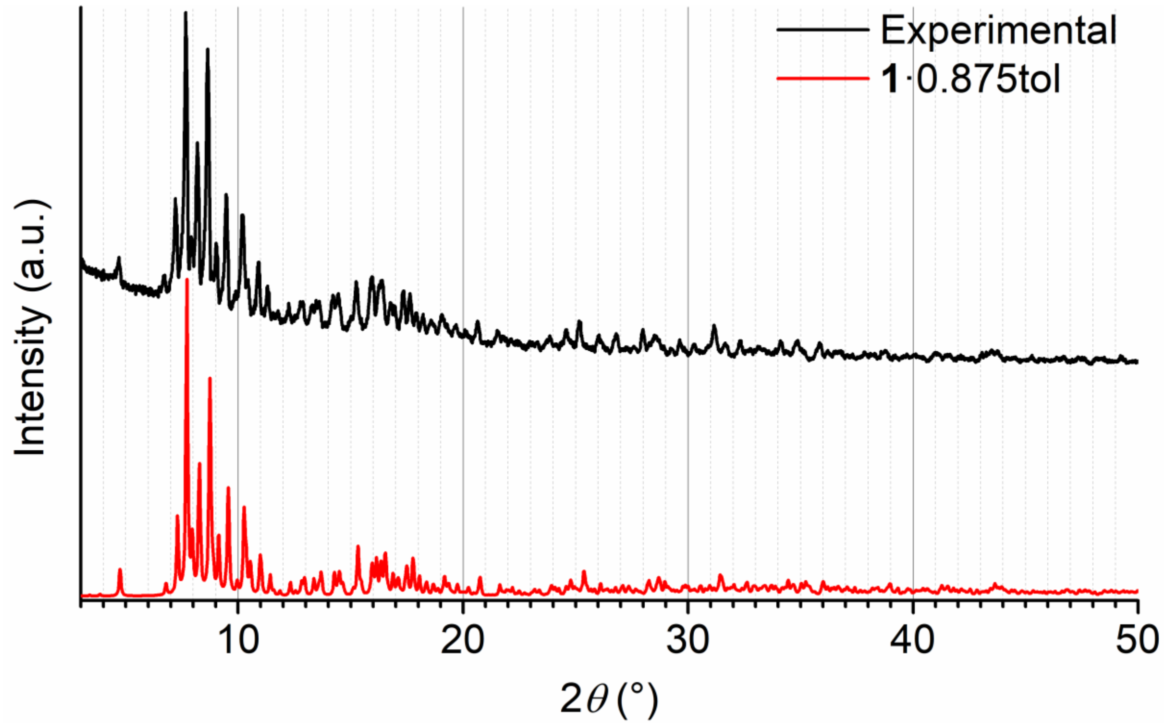

Figure S1. Experimental PXRD pattern of freshly-crystallized material at 298 K (black line). The red line represents the simulated PXRD pattern of **1**·0.875tol, calculated from its SXRD structure collected at 200 K (CCDC 2473728). The 2θ-shift of calculated peak positions for **1**·0.875tol (+0.09° at 2θ = ~8.6°; +0.27° at 2θ = ~31.2°) is due to low-temperature lattice contraction.

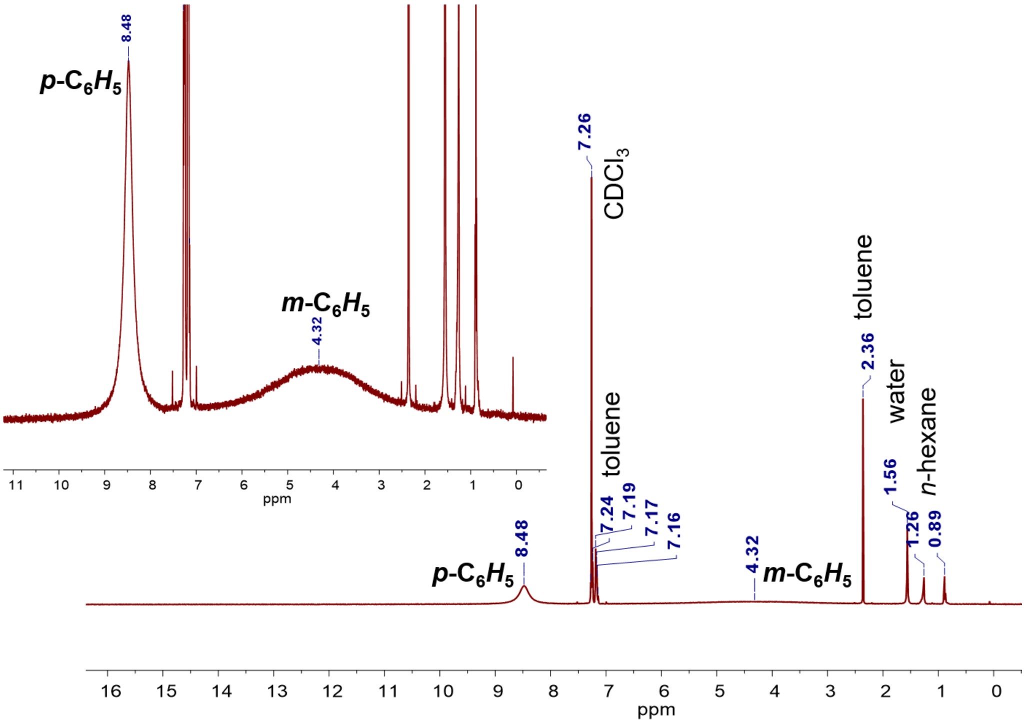

Figure S2. ^{1}H NMR spectrum of **1**·0.3tol·0.1hex in $CDCl_3$. Toluene and *n*-hexane are residuals from crystallization and crystal washing, respectively; water is contained in $CDCl_3$.

2. X-ray crystallography

Table S1. Crystal data and refinement parameters for **1**·0.875tol, α-**1**·0.5tol, and β-**1**·0.5tol.

	**1**·0.875tol	α-**1**·0.5tol	β-**1**·0.5tol
Radiation	Mo-Kα	Mo-Kα	Mo-Kα
λ (Å)	0.71073	0.71073	0.71073
Chemical formula	$C_{34.12}H_{27}O_5PtS_4V$	$C_{31.50}H_{24}O_5PtS_4V$	$C_{31.50}H_{24}O_5PtS_4V$
Formula weight	891.33	856.78	856.78
T (K)	200(2)	200(2)	200(2)
Crystal size (mm^3)	0.21 × 0.07 × 0.01	0.19 × 0.16 × 0.09	0.49 × 0.20 × 0.10
Crystal system	triclinic	monoclinic	triclinic
Space group	$P\bar{1}$	$C2/c$	$P\bar{1}$
a (Å)	11.1670(7)	15.4626(3)	11.1201(3)
b (Å)	23.5720(19)	16.4258(3)	13.6788(4)
c (Å)	26.303(2)	25.7047(5)	21.6462(6)
α (deg)	81.433(3)	90	89.6273(12)
β (deg)	88.573(3)	97.7395(9)	75.8549(11)
γ (deg)	79.106(3)	90	79.4214(12)
V ($Å^3$)	6722.9(9)	6469.1(2)	3135.96(15)
Z	8	8	4
ρ_{calcd} (g cm^{-3})	1.761	1.759	1.815
$2\theta_{min}/2\theta_{max}$ (deg)	3.71/52.13	7.04/55.96	5.02/57.09
Refls. collected/independent	86117/26285	31512/7776	51322/15844
No. of parameters/restraints	1699/327	405/12	722/15
$R1/wR2$ (all data)	0.1144/0.1130	0.0340/0.0683	0.0361/0.0662
$R1/wR2$ ($I > 2\sigma(I)$)	0.0472/0.0876	0.0260/0.0655	0.0256/0.0606
GOF	1.008	1.052	1.070
Largest diff. peak/hole ($eÅ^{-3}$)	0.925/−1.134	1.150/−0.928	1.162/−1.236

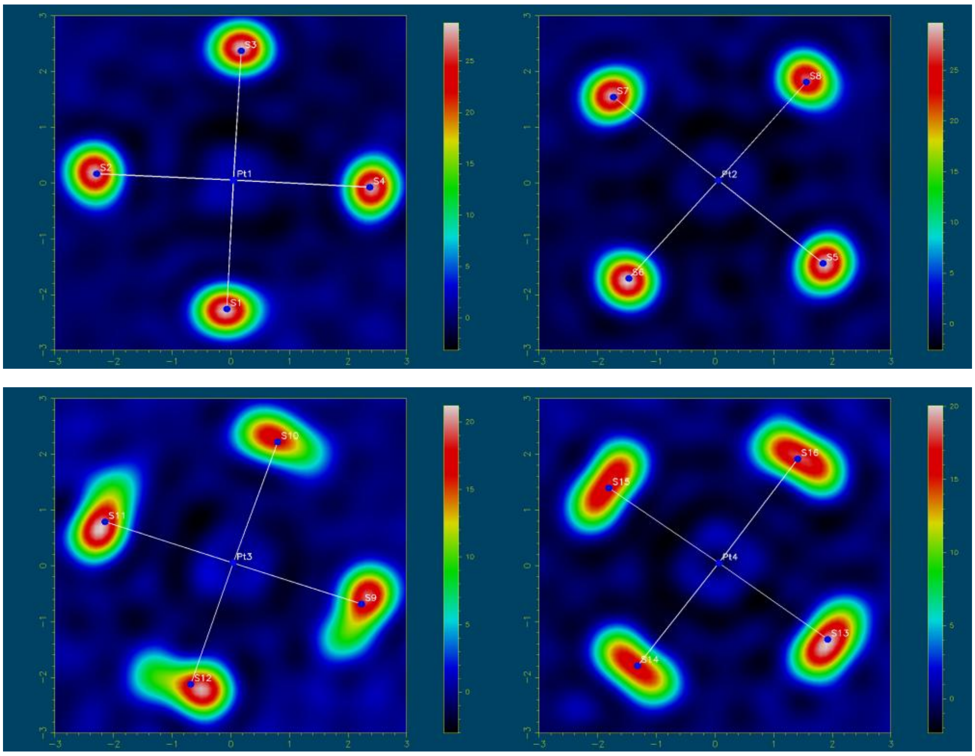

Figure S3. Slant-plane Fourier maps taken in the mean planes of the four PtS_4 moieties of **1**·0.875tol (phases based on isotropic refinement).

3. DFT calculations

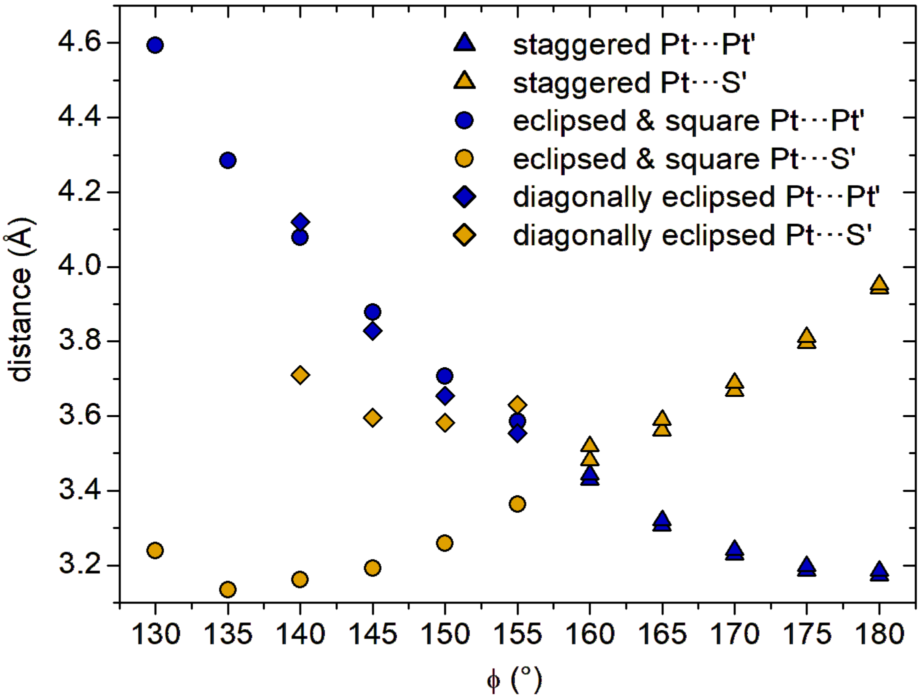

Figure S4. Interatomic distances at the (local) minima of the energy profiles in **Figure 7**.

4. Statistical analysis

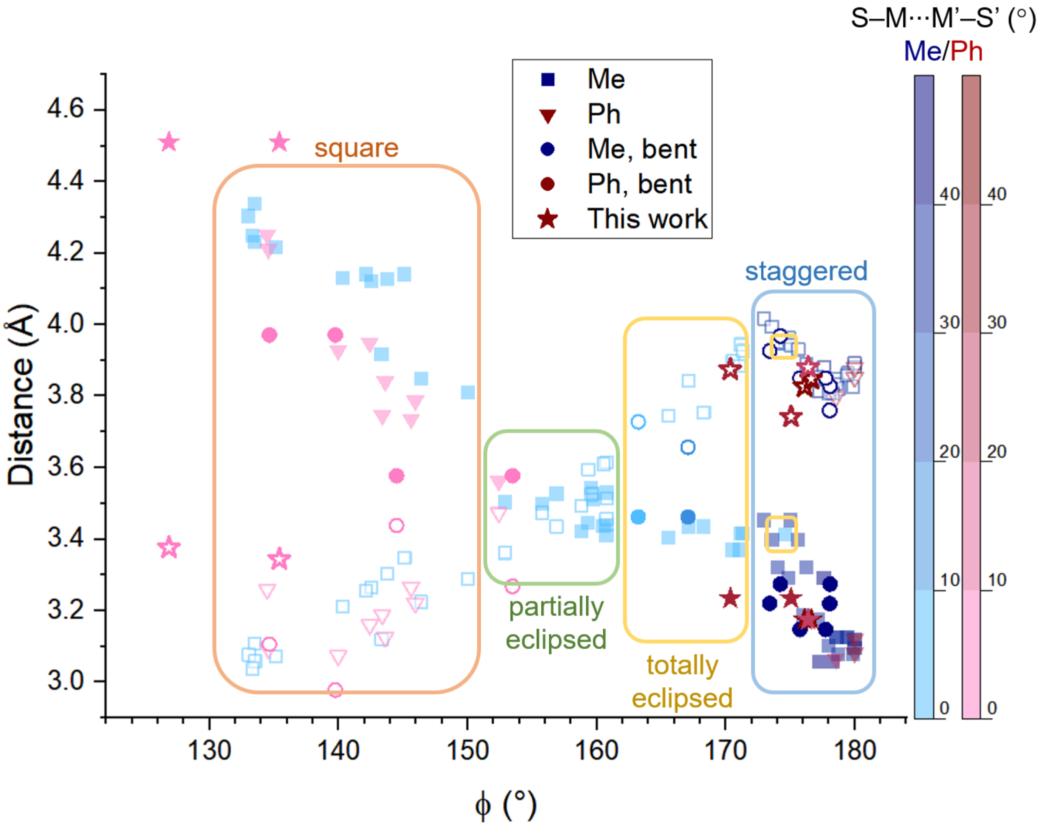

Figure S5. **Figure 8** with the addition of *bent* dimers. Filled symbols represent M···M' distances, empty symbols represent M···S' distances.

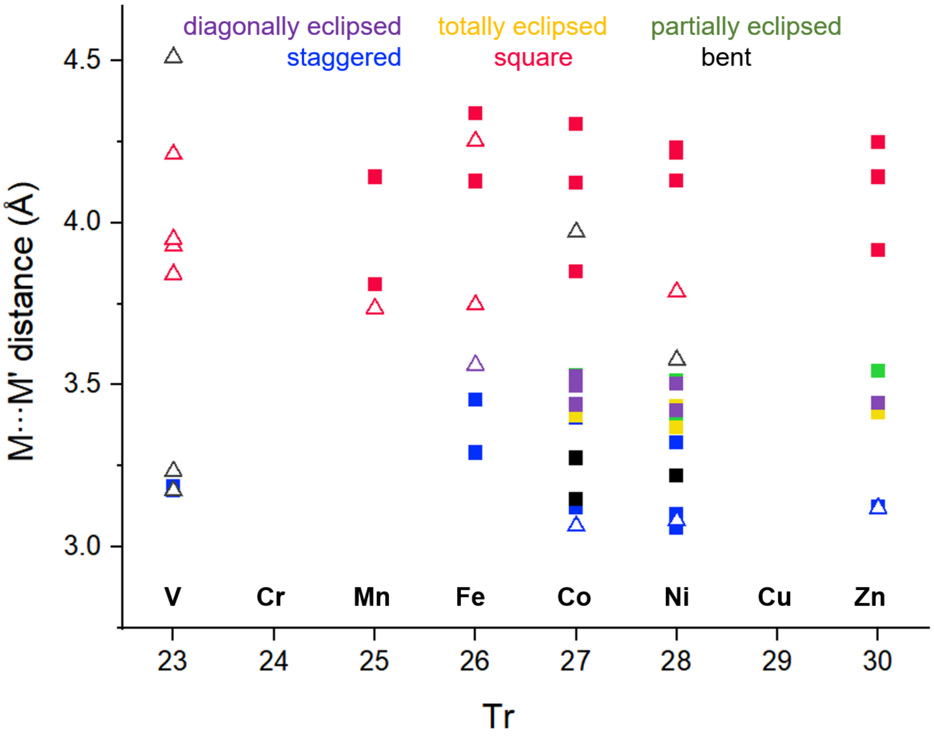

Figure S6. M···M' distance and dimerization mode plotted vs. the nature of the first-row transition metal. Squares correspond to R = Me, triangles to R = Ph.

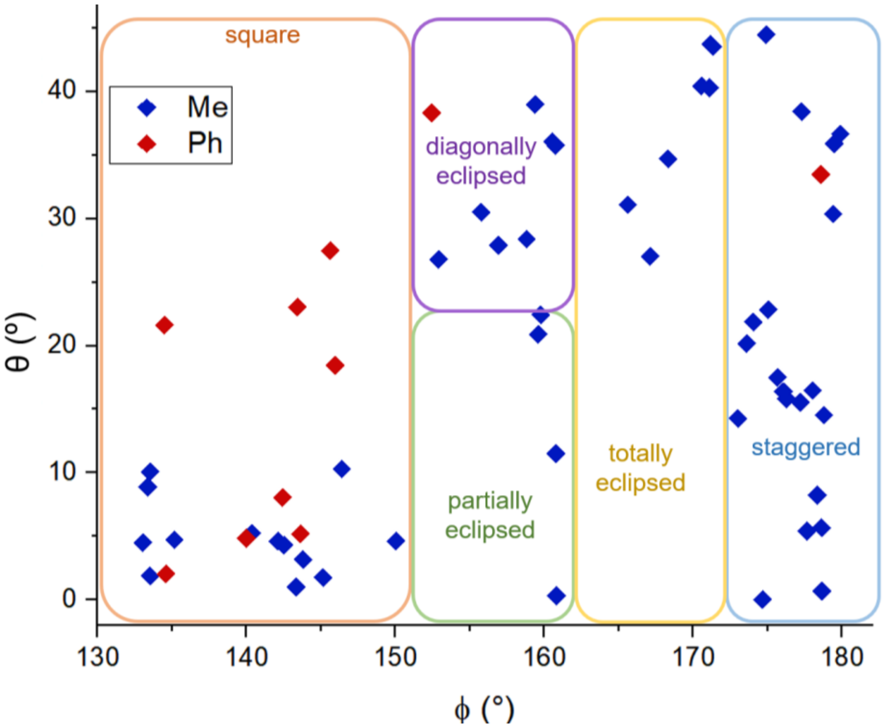

Figure S7. Angle between the (TrMS) and (TrMM'Tr') planes (corresponding approximately to θ) plotted against the Tr–M···M' angle. Data according to CCDC 2025.2.0.[1]

Table S2. Selected geometrical parameters in [MTr(SCOR)$_4$L] dimers with M···M' and M···S' ≤ 5 Å, according to CCDC 2025.2.0[1] and other sources.

Refcode	Tr	M	Axial ligand	Distances (Å)			Angles (°)				Dimer type	Count
				Tr···M	M···M'	M···S'	ϕ	Min torsion S–M···M'–S'	γ	Min θ		
**R = Me**												
ASUFOL	Co	Pt	NCS···{Na(15-crown-5)}	2.664	3.527	3.436	156.889	0.488	0	27.909	diagonally eclipsed	1
ASUFOL	Co	Pt	NCS···{Na(15-crown-5)}	2.655	3.53	3.514	160.809	-0.415	0	0.357	partially eclipsed	2
ASUFUR	Ni	Pt	NCS···{Na(15-crown-5)}	2.601	3.512	3.529	159.74	0.193	0	22.435	partially eclipsed	3
ASUGAY	Zn	Pt	NCS···{Na(15-crown-5)}	2.692	3.543	3.525	159.586	0.287	0	20.916	partially eclipsed	4
ASUGEC	Co	Pt	NCS	2.669	3.404	3.745	165.58	0.773	0	31.098	totally eclipsed	5
ASUGEC	Co	Pt	NCS	2.669	3.412	3.947	174.639	0.444	0	0.022	totally eclipsed	6
ASUGIG	Ni	Pt	NCS	2.616	3.37	3.9	170.548	–0.524	0.532	40.445	totally eclipsed	7
ASUGIG	Ni	Pt	NCS	2.614	3.37	3.885	171.078	–0.524	0.532	40.297	totally eclipsed	7
ASUGOM	Zn	Pt	NCS	2.703	3.416	3.945	171.177	-0.271	0.169	43.693	totally eclipsed	8
ASUGOM	Zn	Pt	NCS	2.708	3.416	3.927	171.313	-0.271	0.169	43.512	totally eclipsed	8
BEYNEC	Ni	Pd	*m*-pyNO_2	2.594	3.503	3.362	152.879	0.284	0	26.811	diagonally eclipsed	9
BEYNIG	Co	Pd	*m*-pyNO_2	2.64	3.498	3.474	155.76	0.861	0	30.507	diagonally eclipsed	10
BEYPAA	Ni	Pt	*m*-pyNO_2	2.587	3.421	3.494	158.802	0.294	0	28.397	diagonally eclipsed	11
COVGAZ	Fe	Pt	py	2.661	4.338	3.109	133.492	-0.645	0	1.903	square	12
GIVLEG	Ni	Pt	DABCO[a]	2.605	3.095	3.882	180	-43.62	0	-	staggered	13
GOMXAK	Mn	Pt	*p*-pySMe	2.706	3.81	3.29	150.039	-0.353	0	4.648	square	14
GOMXEO	Fe	Pt	*p*-pySMe	2.663	3.453	4.017	172.986	36.75	4.33	14.297	staggered	15
GOMXEO	Fe	Pt	*p*-pySMe	2.673	3.291	3.965	174.89	39.277	4.282	44.47	staggered	16
GOMXEO	Fe	Pt	*p*-pySMe	2.676	3.453	3.943	175.016	-36.75	4.33	22.859	staggered	15
GOMXEO	Fe	Pt	*p*-pySMe	2.685	3.291	3.882	177.622	39.277	4.282	5.419	staggered	16
GOMXIS	Co	Pt	*p*-pySMe	2.634	3.398	3.994	173.587	-37.591	4.087	20.183	staggered	17
GOMXIS	Co	Pt	*p*-pySMe	2.643	3.274	3.968	174.225	41.317	5.221	32.284	bent	18
GOMXIS	Co	Pt	*p*-pySMe	2.648	3.398	3.931	175.639	37.591	4.087	17.519	staggered	17
GOMXIS	Co	Pt	*p*-pySMe	2.657	3.274	3.828	178.086	41.317	5.221	15.211	bent	18
GOMXOY	Ni	Pt	*p*-pySMe	2.594	3.22	3.926	173.407	-42.876	6.446	23.322	bent	19
GOMXOY	Ni	Pt	*p*-pySMe	2.592	3.321	3.949	174.019	-37.476	4.084	21.866	staggered	20
GOMXOY	Ni	Pt	*p*-pySMe	2.606	3.321	3.89	176.251	-37.476	4.084	15.845	staggered	20

Refcode	Tr	M	Axial ligand	Distances (Å)			Angles (°)				Dimer type	Count
				Tr···M	M···M'	M···S'	ϕ	Min torsion S–M···M'–S'	γ	Min θ		
GOMXOY	Ni	Pt	*p*-pySMe	2.617	3.22	3.76	178.057	-43.876	6.446	23.302	bent	19
GOMXUE	Zn	Pt	*p*-pySMe	2.617	3.916	3.12	143.318	0.165	0	1.05	square	21
GOMYAL	Mn	Pt	*p*-pyNH_2	2.73	4.141	3.348	145.114	0.922	0	1.79	square	22
GOMYEP	Fe	Pt	*p*-pyNH_2	2.679	4.128	3.304	143.783	0.52	0	3.201	square	23
HOSDEC	Co	Pt	DABCO[a]	2.634	3.120	3.892	180	-44.19	0	-	staggered	24
ITITOX	Co	Pt	*p*-pyMe	2.662	3.147	3.85	175.744	-42.955	5.879	18.827	bent	25
ITITOX	Co	Pt	*p*-pyMe	2.66	3.147	3.85	177.744	-42.955	5.879	25.791	bent	25
NIQZOE	Co	Pt	H_2O	2.634	3.126	3.848	178.609	-36.266	1.727	0.702	staggered	26
NIQZOE	Co	Pt	H_2O	2.624	3.126	3.865	179.447	-36.266	1.727	35.883	staggered	26
NIQZUK	Ni	Pt	H_2O	2.585	3.079	3.821	178.76	-36.323	1.373	14.545	staggered	27
NIQZUK	Ni	Pt	H_2O	2.571	3.079	3.827	179.865	-36.323	1.373	36.683	staggered	27
NIRBAT	Zn	Pt	H_2O	2.648	3.125	3.834	178.607	-35.457	1.897	5.679	staggered	28
NIRBAT	Zn	Pt	H_2O	2.631	3.125	3.859	179.384	-35.457	1.897	30.369	staggered	28
NIRBEX	Co	Pt	*m*-pyNO_2	2.635	3.439	3.615	160.757	0.535	0	35.749	diagonally eclipsed	29
NIRBEX01	Co	Pt	*m*-pyNO_2	2.627	3.437	3.61	160.527	0.565	0	36.062	diagonally eclipsed	30
NIRBIB	Ni	Pt	*m*-pyNO_2	2.562	3.058	3.813	177.262	42.838	2.949	38.434	staggered	31
NIRBIB	Ni	Pt	*m*-pyNO_2	2.568	3.058	3.808	178.33	42.838	2.949	8.254	staggered	31
NIRBOH	Zn	Pt	*m*-pyNO_2	2.628	3.445	3.595	159.355	0.446	0	38.989	diagonally eclipsed	32
OZUWUE	Ni	Pt	H_2O	2.575	3.435	3.843	167.103	6.104	4.8	27.043	totally eclipsed	33
OZUWUE	Ni	Pt	H_2O	2.583	3.435	3.755	168.301	6.104	4.8	34.716	totally eclipsed	33
PIVGUY	Ni	Pt	DMF	2.557	4.217	3.072	135.138	0.79	0	4.756	square	34
PIVTEV	Co	Pt	py	2.63	4.304	3.077	133.027	0.304	0	4.515	square	35
PIVTIZ	Ni	Pt	py	2.583	4.231	3.059	133.507	-0.107	0	10.09	square	36
PIVTOF	Zn	Pt	py	2.618	4.249	3.038	133.348	-0.586	0	8.898	square	37
PIVTUL	Co	Pt	*p*-pyNH_2	2.641	4.122	3.265	142.506	0.329	0	4.326	square	38
PIVVAT	Ni	Pt	*p*-pyNH_2	2.595	4.13	3.212	140.341	0.062	0	5.253	square	39
PIVVEX	Zn	Pt	*p*-pyNH_2	2.662	4.141	3.256	142.101	0.373	0	4.628	square	40
PIVVIB	Co	Pt	DMSO	2.622	3.849	3.225	146.377	-0.207	0	10.321	square	41
QUWDOE	V	Pt	O	2.863	3.175	3.853	177.168	32.222	0.82	15.553	staggered	42
TORHUG	Ni	Pt	H_2O	2.581	3.411	3.457	160.769	0.031	0	11.524	partially eclipsed	43
ZUVRER[b]	Ni	Pt	*m*-pyNO_2	2.573	3.138	3.822	177.661	-34.68	2.758	17.378	staggered	
ZUVRER01[b]	Ni	Pt	*m*-pyNO_2	2.573	3.12	3.818	177.862	-33.571	2.68	16.821	staggered	
ZUVRER02[b]	Ni	Pt	*m*-pyNO_2	2.573	3.142	3.824	177.613	35.087	2.794	17.582	staggered	
ZUVRER03[b]	Ni	Pt	*m*-pyNO_2	2.572	3.132	3.821	177.699	34.407	2.715	17.24	staggered	

Refcode	Tr	M	Axial ligand	Distances (Å)			Angles (°)				Dimer type	Count
				Tr···M	M···M'	M···S'	ϕ	Min torsion S–M···M'–S'	γ	Min θ		
ZUVRER04[b]	Ni	Pt	*m*-pyNO$_2$	2.569	3.112	3.812	177.955	33.417	2.676	16.741	staggered	
ZUVRER05[b]	Ni	Pt	*m*-pyNO$_2$	2.573	3.148	3.828	177.586	-35.411	2.809	17.744	staggered	
ZUVRER06[b]	Ni	Pt	*m*-pyNO$_2$	2.571	3.122	3.816	177.807	-33.85	2.684	16.961	staggered	
ZUVRER07[b]	Ni	Pt	*m*-pyNO$_2$	2.573	3.107	3.812	177.982	33.004	2.664	16.532	staggered	
ZUVRER08[b]	Ni	Pt	*m*-pyNO$_2$	2.571	3.11	3.811	177.946	-33.198	2.681	16.631	staggered	
ZUVRER09[b]	Ni	Pt	*m*-pyNO$_2$	2.571	3.127	3.817	177.747	34.067	2.724	17.071	staggered	
ZUVRER10[b]	Ni	Pt	*m*-pyNO$_2$	2.572	3.102	3.809	178.007	-32.887	2.71	16.474	staggered	44
2443816[c]	V	Pd	O	2.911	3.187	3.840	176.05	-32.17	1.16	16.40	staggered	45
**R = Ph**												
COVGED	Fe	Pt	py	2.645	3.562	3.474	152.407	0.717	0	38.318	diagonally eclipsed	1
GALVAS	Fe	Pt	H_2O	2.632	4.251	3.26	134.464	0.57	0	21.627	square	2
GALVEW	Co	Pt	H_2O	2.599	3.065	3.799	178.56	23.664	2.368	33.458	staggered	3
GALVIA	Co	Pt	H_2O	2.552	3.971	3.106	134.634	1.873	7.848	14.264	bent	4
GALVIA	Co	Pt	H_2O	2.567	3.971	2.978	139.74	1.873	7.848	11.747	bent	4
GALVOG	Ni	Pt	H_2O	2.565	3.082	3.852	180	-39.769	0	-	staggered	5
ICOHER	Mn	Pt	*p*-pySMe	2.702	3.736	3.266	145.612	0.233	0	27.479	square	6
ICOHIV	Fe	Pt	*p*-pySMe	2.654	3.747	3.189	143.394	-0.215	0	23.046	square	7
JANCOT	Zn	Pt	H_2O	2.634	3.12	3.88	180	-39.734	0	-	staggered	8
QUWDUK	V	Pt	O	2.782	3.841	3.126	143.616	-0.291	0	5.213	square	9
VOYCIX	Ni	Pt	Cl	2.594	3.787	3.221	145.951	0.933	0	18.472	square	10
ZUVPOZ	Ni	Pt	*m*-pyNO$_2$	2.551	3.577	3.438	144.485	2.942	11.896	34.562	bent	11
ZUVPOZ	Ni	Pt	*m*-pyNO$_2$	2.551	3.577	3.268	153.472	2.942	11.896	38.91	bent	11
2443817[c]	V	Pd	O	2.836	3.928	3.074	139.973	0	0	4.859	square	12
2443818[c]	V	Pd	O	2.838	4.211	3.094	134.557	0	0	2.058	square	13
2490019	V	Pt	O	2.798	3.949	3.161	142.41	0	0	8.086	square	14
**R = other**												
ZUVPUF[d]	Ni	Pt	*m*-pyNO$_2$	2.549	3.52	3.617	159.348	0.215	0	30.887	diagonally eclipsed	
ZUVQAM[e]	Ni	Pt	*m*-pyNO$_2$	2.558	3.332	3.49	159.978	0.049	0	26.783	diagonally eclipsed	
ZUVQAM[e]	Ni	Pt	*m*-pyNO$_2$	2.561	3.462	3.728	163.222	6.812	5.921	19.187	bent	
ZUVQAM[e]	Ni	Pt	*m*-pyNO$_2$	2.578	3.462	3.657	167.082	6.812	5.921	10.818	bent	

[a]1,4-diazabicyclo[2,2,2]octane. [b]Entries for the same crystal collected at different temperatures; only ZUVRER10 (100 K), quoted in the original article, was included in the statistics. [c]From Ref. [2]. [d]R = *t*Bu. [e]R = C_5H_8.

5. References

[1] C. R. Groom, I. J. Bruno, M. P. Lightfoot, S. C. Ward, *Acta Crystallogr. Sect. B Struct. Sci. Cryst. Eng. Mater.* **2016**, *72*, 171–179.

[2] O. Mironova, G. Bellini, A. Nicolini, M. Imperato, A. Ranieri, M. Borsari, M. Briganti, R. Clérac, M. Rouzières, E. Salvadori, M. C. Pagliero, M. Chiesa, A. Cornia, **2025**, ChemRxiv preprint DOI: 10.26434/chemrxiv-2025-l5n9j-v2.
