# Supplementary material for: Multiple Dimerization Modes in Thiocarboxylate Paddlewheel Complexes: A Comprehensive View of Energy Landscapes from DFT Calculations and Statistics
Source: Chemistry. 2025 Dec 2;32(15):e03004. doi: 10.1002/chem.202503004 (PMC13107499; doi:10.1002/chem.202503004)
Supplement: Supplementary file 2 — Supporting File 2: chem70500‐sup‐0002‐DataFile.zip. [file CHEM-32-e03004-s002.zip › DFT optimized structures.zip!/thermochemical data of optimized structures.pdf]

name	initial xyz	ORCA input details	electronic energy (E_h)	ΔE_{int} (kcal/mol)	dispersion (E_h)	ΔE_{int}^{disp} (kcal/mol)	G (kcal/mol)	ΔG_{int} (kcal/mol)
**PBE0 def2-TZVPP D3BJ H-only optimization in the gas phase**								
**PtVOMe**	XRD	TIGHTSCF TIGHTOPT DEFGRID3	**-3343.54319414846**	**-14.840**	-0.054547640	**-14.241**		
**PtVOMe_dimer_T**	XRD	TIGHTSCF TIGHTOPT DEFGRID3	**-6687.11003684945**		-0.131789210			
**PtVOPhCH2Cl2**	XRD	TIGHTSCF TIGHTOPT DEFGRID3	**-4109.89948600415**	**-15.549**	-0.101619489	**-15.799**		
**PtVOPhCH2Cl2_dimer_T**	XRD	TIGHTSCF TIGHTOPT DEFGRID3	**-8219.82375162745**		-0.228416199			
**PtVOPh7s8tol_mol1**	XRD	TIGHTSCF TIGHTOPT DEFGRID3	**-4109.88911582453**	**-18.298**	-0.101262662	**-17.071**		
**PtVOPh7s8tol_mol2**	XRD	TIGHTSCF TIGHTOPT DEFGRID3	**-4109.88803680654**		-0.101272201			
**PtVOPh7s8tol_dimer_T**	XRD	TIGHTSCF TIGHTOPT DEFGRID3	**-8219.80631192695**		-0.229738634			
**PBE0 def2-TZVPP D3BJ full optimization in the gas phase**								
**PtVOMe_gasphase**	XRD	TIGHTSCF TIGHTOPT DEFGRID3	**-3343.54637213195**	**-15.554**	-0.054849852	**-14.187**	**-3343.39470744**	**0.463**
**PtVOMe_dimer_T_gasphase**	XRD	TIGHTSCF TIGHTOPT DEFGRID3	**-6687.11753076897**		-0.132308336		**-6686.78867733**	
**PtVOPhCH2Cl2_gasphase**	XRD	TIGHTSCF TIGHTOPT DEFGRID3	**-4109.90712873014**	**-16.574**	-0.102130934	**-17.225**	**-4109.55281245**	**-0.680**
**PtVOPhCH2Cl2_dimer_T_gasphase**	XRD	TIGHTSCF TIGHTOPT DEFGRID3*	**-8219.84067038288**		-0.231711216		**-8219.10670908**	
**PtVOPh7s8tol_mol1_gasphase**	XRD	TIGHTSCF TIGHTOPT DEFGRID3	**-4109.90710336140**	**-18.598**	-0.102033884	**-17.899**	**-4109.55285277**	**-2.328**
**PtVOPh7s8tol_mol2_gasphase**	XRD	TIGHTSCF TIGHTOPT DEFGRID3	**-4109.90710650307**		-0.102105011		**-4109.55280238**	
**PtVOPh7s8tol_dimer_T_gasphase**	XRD	TIGHTSCF TIGHTOPT DEFGRID3*	**-8219.84384759421**		-0.232663166		**-8219.10936426**	
**PBE0 def2-TZVPP D3BJ full optimization in solvent [CPCM(toluene)]**								
**PtVOMe_solv**	XRD	TIGHTSCF TIGHTOPT DEFGRID3	**-3343.55611744621**	**-15.769**	-0.054817391	**-14.170**	**-3343.40500952**	**0.266**
**PtVOMe_dimer_T_solv**	XRD	TIGHTSCF TIGHTOPT DEFGRID3	**-6687.13736456834**		-0.132215953		**-6686.80959474**	
**PtVOPh7s8tol_mol1_solv**	XRD	TIGHTSCF TIGHTOPT DEFGRID3	**-4109.91868894179**	**-18.722**	-0.101996049	**-18.118**	**-4109.56497843**	**-2.434**
**PtVOPh7s8tol_mol2_solv**	XRD	TIGHTSCF TIGHTOPT DEFGRID3	**-4109.91868994790**		-0.102063703		**-4109.56489170**	
**PtVOPh7s8tol_dimer_T_solv**	XRD	TIGHTSCF TIGHTOPT DEFGRID3*	**-8219.86721352213**		-0.232932806		**-8219.13374920**	
*DEFGRID2 in analytical frequency calculations to achieve convergence of CP-SCF equations								
